# Pan-cancer and cross-population genome-wide association studies dissect shared genetic backgrounds underlying carcinogenesis

**DOI:** 10.1038/s41467-023-39136-7

**Published:** 2023-06-20

**Authors:** Go Sato, Yuya Shirai, Shinichi Namba, Ryuya Edahiro, Kyuto Sonehara, Tsuyoshi Hata, Mamoru Uemura, the Biobank Japan Project, Yuji Yamanashi, Yoichi Furukawa, Takayuki Morisaki, Yoshinori Murakami, Yoichiro Kamatani, Kaori Muto, Akiko Nagai, Wataru Obara, Ken Yamaji, Kazuhisa Takahashi, Satoshi Asai, Yasuo Takahashi, Takao Suzuki, Nobuaki Sinozaki, Hiroki Yamaguchi, Shiro Minami, Shigeo Murayama, Kozo Yoshimori, Satoshi Nagayama, Daisuke Obata, Masahiko Higashiyama, Akihide Masumoto, Yukihiro Koretsune, Koichi Matsuda, Yuichiro Doki, Hidetoshi Eguchi, Yukinori Okada

**Affiliations:** 1 Department of Statistical Genetics, Osaka University Graduate School of Medicine, Suita, Japan; 2 Department of Gastroenterological Surgery, Graduate School of Medicine, Osaka University, Osaka, Japan; 3 Department of Respiratory Medicine and Clinical Immunology, Osaka University Graduate School of Medicine, Suita, Japan; 4 Laboratory of Statistical Immunology, Immunology Frontier Research Center (WPI-IFReC), Osaka University, Suita, Japan; 5 Laboratory for Systems Genetics, RIKEN Center for Integrative Medical Sciences, Yokohama, Japan; 6 Department of Genome Informatics, Graduate School of Medicine, the University of Tokyo, Tokyo, Japan; 7 Laboratory of Clinical Genome Sequencing, Department of Computational Biology and Medical Sciences, Graduate School of Frontier Sciences, the University of Tokyo, Tokyo, Japan; 8 Division of Genetics, The Institute of Medical Science, The University of Tokyo, Tokyo, Japan; 9 Division of Clinical Genome Research, Institute of Medical Science, The University of Tokyo, Tokyo, Japan; 10 Division of Molecular Pathology, IMSUT Hospital Department of Internal Medicine, Institute of Medical Science, The University of Tokyo, Tokyo, Japan; 11 Department of Cancer Biology, Institute of Medical Science, The University of Tokyo, Tokyo, Japan; 12 Laboratory of Complex Trait Genomics, Department of Computational Biology and Medical Sciences, Graduate School of Frontier Sciences, The University of Tokyo, Tokyo, Japan; 13 Department of Public Policy, Institute of Medical Science, The University of Tokyo, Tokyo, Japan; 14 Department of Urology, Iwate Medical University, Iwate, Japan; 15 Department of Internal Medicine and Rheumatology, Juntendo University Graduate School of Medicine, Tokyo, Japan; 16 Department of Respiratory Medicine, Juntendo University Faculty of Medicine and Graduate School of Medicine, Tokyo, Japan; 17 Division of Pharmacology, Department of Biomedical Science, Nihon University School of Medicine, Tokyo, Japan; 18 Division of Genomic Epidemiology and Clinical Trials, Clinical Trials Research Center, Nihon University School of Medicine, Tokyo, Japan; 19 Tokushukai Group, Tokyo, Japan; 20 Department of Hematology, Nippon Medical School, Tokyo, Japan; 21 Department of Bioregulation, Nippon Medical School, Kawasaki, Japan; 22 Tokyo Metropolitan Geriatric Hospital and Institute of Gerontology, Tokyo, Japan; 23 Fukujuji Hospital, Japan Anti-Tuberculosis Association, Tokyo, Japan; 24 The Cancer Institute Hospital of the Japanese Foundation for Cancer Research, Tokyo, Japan; 25 Center for Clinical Research and Advanced Medicine, Shiga University of Medical Science, Shiga, Japan; 26 Department of General Thoracic Surgery, Osaka International Cancer Institute, Osaka, Japan; 27 Iizuka Hospital, Fukuoka, Japan; 28 National Hospital Organization Osaka National Hospital, Osaka, Japan

**Keywords:** Genome-wide association studies, Cancer genetics, Cancer genetics

## Abstract

Integrating genomic data of multiple cancers allows de novo cancer grouping and elucidating the shared genetic basis across cancers. Here, we conduct the pan-cancer and cross-population genome-wide association study (GWAS) meta-analysis and replication studies on 13 cancers including 250,015 East Asians (Biobank Japan) and 377,441 Europeans (UK Biobank). We identify ten cancer risk variants including five pleiotropic associations (e.g., rs2076295 at *DSP* on 6p24 associated with lung cancer and rs2525548 at *TRIM4* on 7q22 nominally associated with six cancers). Quantifying shared heritability among the cancers detects positive genetic correlations between breast and prostate cancer across populations. Common genetic components increase the statistical power, and the large-scale meta-analysis of 277,896 breast/prostate cancer cases and 901,858 controls identifies 91 newly genome-wide significant loci. Enrichment analysis of pathways and cell types reveals shared genetic backgrounds across said cancers. Focusing on genetically correlated cancers can contribute to enhancing our insights into carcinogenesis.

## Abstract (web summary)

Investigating genetically correlated cancers can enhance our understanding of carcinogenesis. Here, the authors perform Pan-cancer and cross-population GWAS meta-analysis to identify novel cancer risk loci and highlight shared heritability between breast and prostate cancer.

## Introduction

Cancer is a leading cause of death worldwide, it kills 10 million people every year, and a rapidly growing burden^1^. As well as environmental and somatic factors, hereditary components play an important role in cancer development. Genome-wide association studies (GWAS) have detected hundreds of genetic variants associated with specific cancer risk and pleiotropic associations with different cancers, including 2q33 (*CASP8-ALS2CR12*)^2,3^, 5p15 (*TERT-CLPTM1L*)^4,5^ and 8q24^6,7^. Analyzing genomic data of multiple cancers to elucidate pleiotropy and genetic correlations provides opportunities for de novo cancer grouping and highlighting the common mechanisms underlying cancer etiology^8^.

A previous cross-cancer GWAS meta-analysis of five cancers using data from consortia such as the Genetic Associations and Mechanisms in Oncology (GAME-ON) Network, has applied a subset-based approach^9^ and identified a pleiotropic association at 1q22 involving breast and lung squamous cell carcinoma^10^. Previous studies have estimated genetic correlations among cancer pairs and detected genetically correlated cancer pairs. One study including 13 cancers found four pairs with marginally significant correlations^11^. Lindström et al. found significant correlations between pancreatic and colorectal cancer, and lung and colorectal cancer^12^. In another study conducted by Jiang et al., five cancer pairs showed significant correlations (lung-head/neck, colorectal-lung, breast-ovarian, breast-lung, and breast-colorectal)^13^. A recent study evaluated genetic correlations across 18 cancers from two large cohorts of European ancestry and revealed shared heritability between 12 cancer pairs, of which only colon and rectal cancers were genetically correlated after multiple testing corrections^14^. While these studies have improved our knowledge of cancer susceptibility, most of them have been undertaken in Europeans. Despite the significant differences in the incidence of certain cancers across populations (for example, the higher incidence of hepatocellular carcinoma in East Asians^1^), the geographical distribution of heritable cancer risk remains unclear. To investigate the global landscape of human cancer genetics, a large-scale genomic analysis across cancers and populations is required.

Here, we conducted the pan-cancer and cross-population GWAS meta-analysis and replication studies on 13 cancers, examining 250,015 East Asians including 61,465 cancer cases from the BioBank Japan (BBJ)^15^ and 377,441 Europeans including 43,098 cancer cases from the UK Biobank (UKB)^16^. A standard fixed-effects approach detected ten loci newly satisfying the genome-wide significance threshold (*P* < 5.0 × 10^−8^), of which five showed general associations across cancers. We then estimated genetic correlations among the cancers and identified significant positive genetic correlations between breast and prostate cancer both in BBJ and UKB. This relationship was independently validated in FinnGen^17^, a Finnish biobank, and the large-scale meta-analysis including the largest-to-date GWAS datasets of the two cancers identified 91 newly genome-wide significant loci (*n*_Case_ = 277,896, *n*_Control_ = 901,858). Further enrichment analysis of pathways and cell types demonstrated shared genetic backgrounds across both cancers. Our comprehensive genetic study offers further insights into the complex biology underlying carcinogenesis.

## Results

### Pan-cancer and cross-population GWAS meta-analysis

**Fig. 1:**
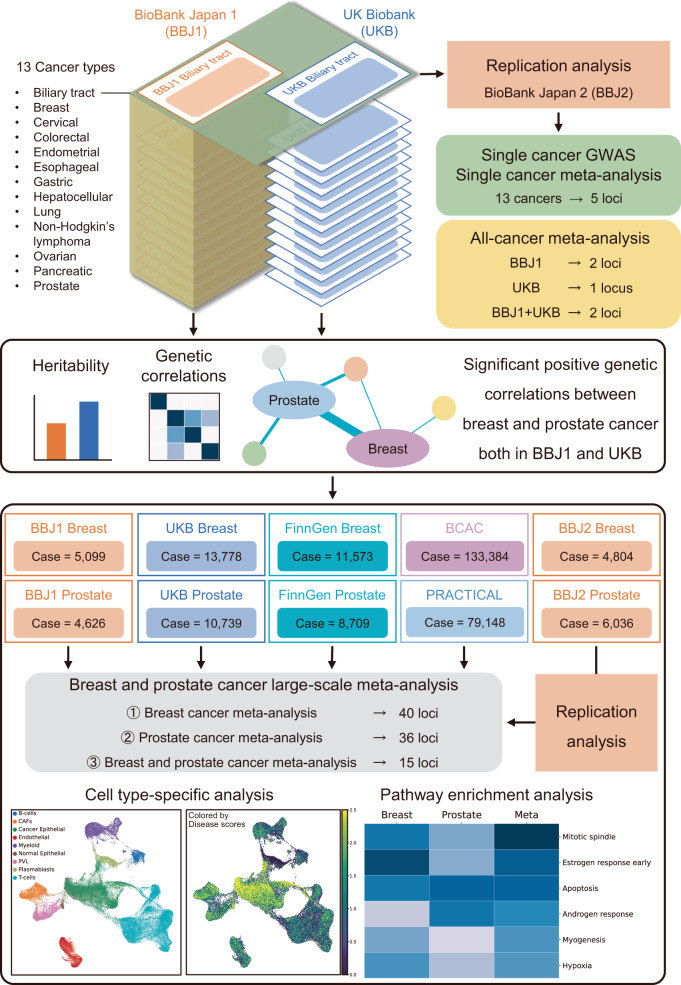
The study overview. The pan-cancer and cross-population GWAS meta-analysis on 13 cancer types followed by the replication analysis examining 250,015 East Asians from the BioBank Japan (BBJ) and 377,441 Europeans from the UK Biobank (UKB) identified ten loci newly satisfying the genome-wide significance threshold (upper). We then estimated the heritability and genetic correlations among the cancers and found significant positive genetic correlations between breast and prostate cancer both in BBJ and UKB (middle). The breast and prostate cancer large-scale meta-analysis including the FinnGen datasets, and the largest-to-date GWAS datasets of breast (BCAC) and prostate cancer (PRACTICAL) detected 91 newly genome-wide significant loci. Further enrichment analysis of cell types and pathways demonstrated shared genetic backgrounds between the two cancers (bottom).

An overview of this study is presented in Fig. 1. Patients diagnosed with any of the following 13 cancers including biliary tract, breast, cervical, colorectal, endometrial, esophageal, gastric, hepatocellular, lung, non-Hodgkin’s lymphoma, ovarian, pancreatic, and prostate cancer from the two biobank resources (BBJ for Japanese and UKB for Europeans) were enrolled in this study. We enrolled control subjects without cancer from the respective cohorts. In the discovery GWAS/meta-analysis, the sample sizes were 29,753 cases and 150,462 controls in the BBJ first cohort (BBJ1) and 43,098 cases and 334,343 controls in UKB. We used 31,712 cases and 38,088 controls in the BBJ second cohort (BBJ2) for our replication analysis. The characteristics of each cohort and results of single cancer GWAS in each ancestry are shown in Table 1, Supplementary Figs. 1 and 2, and Supplementary Data 1. While most genome-wide significant loci were previously associated with the cancer of interest^18–22^, we identified two novel loci in the single cancer GWAS in UKB (colorectal: rs143926630 at *TULP4* on 6q25, *P* = 1.3 × 10^−8^; non-Hodgkin’s lymphoma: rs370149412 at *MEF2B* on 19p13, *P* = 4.7 × 10^−8^; Table 2 and Supplementary Fig. 2). Our single cancer GWAS of East Asians and Europeans replicated 216 and 306 signals, respectively, among the 495 previously reported cancer risk variants in GWAS Catalog^18^ (Supplementary Data 3).

**Table 1:** Summary of the single cancer GWAS and cross-population single cancer meta-analysis

Cancer type	Discovery														Replication		
	BioBank Japan 1 (BBJ1) Sample size		λ_GC_	*h*^2^(SE)	GWAS loci	UK Biobank (UKB) Sample size		λ_GC_	*h*^2^(SE)	GWAS loci	New loci	Cross-population			BioBank Japan 2 (BBJ2) Sample size		Additional new loci
	Case	Control				Case	Control					λ_GC_	GWAS loci	New loci	Case	Control	
Biliary tract	232	150,462	1.001	0.032 (0.144)	0	525	334,343	1.001	0.119 (0.110)	0	0	1.011	0	0	584	38,088	0
Breast	5,099	70,676	1.005	0.095 (0.009)	7	13,778	183,466	1.030	0.146 (0.007)	25	0	1.056	33	1	4,804	18,138	0
Cervical	567	70,676	1.003	0.110 (0.031)	1	313	183,466	1.003	0.189 (0.102)	0	0	1.007	1	0	1,358	18,138	0
Colorectal	6,075	150,462	1.020	0.140 (0.011)	14	7,194	334,343	1.022	0.174 (0.015)	10	1	1.048	23	0	7,382	38,088	0
Endometrial	948	70,676	1.010	0.081 (0.027)	0	1,892	183,466	1.009	0.062 (0.026)	1	0	1.016	0	0	1,012	18,138	0
Esophageal	1,159	150,462	0.997	0.206 (0.027)	2	1,387	334,343	1.016	0.206 (0.044)	0	0	1.005	3	0	1,018	38,088	0
Gastric	5,775	150,462	1.021	0.095 (0.009)	4	1,023	334,343	0.999	0.013 (0.054)	0	0	1.030	4	0	4,324	38,088	0
Hepatocellular	1,361	150,462	1.011	0.143 (0.036)	1	339	334,343	0.994	0.190 (0.190)	2	0	1.009	3	0	2,180	38,088	1
Lung	3,486	150,462	1.018	0.136 (0.019)	6	4,255	334,343	1.016	0.293 (0.028)	3	0	1.029	7	1	3,477	38,088	0
Non-Hodgkin’s lymphoma	736	150,462	1.016	0.138 (0.046)	1	1,997	334,343	1.010	0.073 (0.014)	2	1	1.018	5	0	935	38,088	0
Ovarian	685	70,676	1.005	0.023 (0.027)	0	1,446	183,466	1.001	0.006 (0.026)	0	0	1.009	0	0	835	18,138	0
Pancreatic	281	150,462	0.999	0.312 (0.156)	0	1,328	334,343	1.004	0.031 (0.051)	1	0	1.005	1	0	604	38,088	0
Prostate	4,626	79,786	1.024	0.202 (0.009)	20	10,739	150,877	1.021	0.172 (0.007)	39	0	1.051	45	0	6,036	19,950	0

*h*^2^ heritability on the liability scale.

**Table 2:** Summary of the variants newly satisfying the genome-wide significance threshold from the single/all cancer GWAS/meta-analysis

Cancer type	Cohort	Chr:Position	rsID	REF/ALT	Nearest gene	Functional annotation	Discovery								Replication					
							BioBank Japan 1 (BBJ1)			UK Biobank (UKB)			Cross-population		BBJ2		BBJ1 + BBJ2		BBJ1 + UKB + BBJ2	
							EAF	OR	*P*	EAF	OR	*P*	OR	*P*	OR	*P*	OR	*P*	OR	*P*
**Single cancer GWAS/meta-analysis**																				
Breast	BBJ/UKB	1:17719005	rs2800691	C/T	*PADI6*	Intron	0.35	0.93	0.0013	0.27	0.94	7.5 × 10^−6^	0.94	**3.6** × **10**^**−8**^	0.97	0.20	0.95	0.0010	0.94	**3.1** × **10**^**−8**^
Colorectal	UKB	6:158652195	rs143926630	GA/G	*TULP4*	Intergenic	–	–	–	0.26	1.12	**1.3** × **10**^**−8**^	–	–	–	–	–	–	–	–
Hepatocellular	BBJ	12:113117897	rs77753011	G/T	*RPH3A*	Intron	0.08	0.77	8.1 × 10^−7^	–	–	–	–	–	0.78	1.4 × 10^−9^	0.78	**5.5** × **10**^**−15**^	–	–
Lung	BBJ/UKB	6:7563232	rs2076295	T/G	*DSP*	Intron	0.49	1.12	1.4 × 10^−6^	0.45	1.07	0.0018	1.10	**2.6** × **10**^**−8**^	1.10	1.7 × 10^−4^	1.11	1.1 × 10^−9^	1.10	**1.8** × **10**^**−11**^
NHL	UKB	19:19269254	rs370149412	TAA/T	*MEF2B*	Intron	–	–	–	0.46	1.20	**4.7** × **10**^**−8**^	–	–	–	–	–	–	–	–
**All-cancer meta-analysis**																				
All-cancer	BBJ	1:222841614	rs36079339	G/A	*AIDA*	UTR3	0.54	0.95	6.9 × 10^−7^	0.72	1.00	0.90	0.98	6.1 × 10^−4^	0.96	1.0 × 10^−4^	0.96	**3.9** × **10**^**−10**^	–	–
All-cancer	BBJ	3:185508591	rs11927381	T/C	*IGF2BP2*	Intron	0.34	0.95	**2.4** × **10**^**−8**^	0.31	0.99	0.37	0.97	1.9 × 10^−5^	0.97	4.4 × 10^−4^	0.96	**1.3** × **10**^**−10**^	–	–
All-cancer	UKB	1:107566689	rs56111229	T/A	*PRMT6*	Intergenic	0.29	1.00	0.94	0.35	1.05	**2.0** × **10**^**−8**^	1.03	1.3 × 10^−5^	–	–	–	–	–	–
All-cancer	BBJ/UKB	4:148276400	rs2059904	A/G	*EDNRA*	Intergenic	0.32	1.05	2.2 × 10^−6^	0.17	1.02	0.022	1.04	6.9 × 10^−7^	1.03	0.0041	–	–	1.03	**1.2** × **10**^**−8**^
All-cancer	BBJ/UKB	7:99553167	rs2525548	C/G	*AZGP1*	Intergenic	0.35	0.96	1.0 × 10^−5^	0.58	0.97	3.8 × 10^−5^	0.96	**2.5** × **10**^**−9**^	0.99	0.15	–	–	0.97	**5.1** × **10**^**−9**^

All statistical tests are two-sided and not adjusted for multiple comparisons. *P* values satisfying the genome-wide significance threshold are shown in bold.

*Chr* chromosome, *REF* reference allele, *ALT* alternative allele, *EAF* effect allele frequency in the control subjects, *OR* odds ratio, *NHL* Non-Hodgkin’s lymphoma.

First, to enhance the statistical power and detect novel loci satisfying the genome-wide significance (*P* < 5.0 × 10^−8^), a cross-population GWAS meta-analysis of each cancer (single cancer meta-analysis) was conducted (Table 1 and Supplementary Data 2). In each meta-analysis of breast and lung cancer across BBJ1 and UKB, we identified one locus newly satisfying the genome-wide significance threshold (breast: rs2800691 at *PADI6* on 1p36, *P* = 3.6 × 10^−8^; lung: rs2076295 at *DSP* on 6p24, *P* = 2.6 × 10^−8^; Table 2 and Supplementary Fig. 3). Of these, the lead variant associated with breast cancer was correlated with the previously reported breast cancer risk variant, rs1924553 at *PADI6* (*r*^2^ = 0.21 in Europeans; *r*^2^ = 0.42 in East Asians), but the previous study did not detect genome-wide significant associations at the *PADI6* locus (*P* = 5.4 × 10^−7^)^23^. The lead variant of the two loci had the same directional effect across ancestries (Table 2). We investigated the impacts of these cancer-associated variants on the messenger RNA levels in the Genotype-Tissue Expression v8 (GTEx) database^24^ of expression quantitative trait loci (eQTL). Of these, rs2076295, associated with lung cancer, is an intronic variant of *DSP*; the risk allele rs2076295-G has been associated with decreased *DSP* gene expression in lung (Supplementary Fig. 5a). Further analysis using the Simple Sum (SS) method implemented in LocusFocus^25^ supported strong colocalization between the GWAS and eQTL signals (SS *P*-value = 1.0 × 10^−8^; Supplementary Fig. 6a), indicating the potential impact of the variant.

**Fig. 2:**
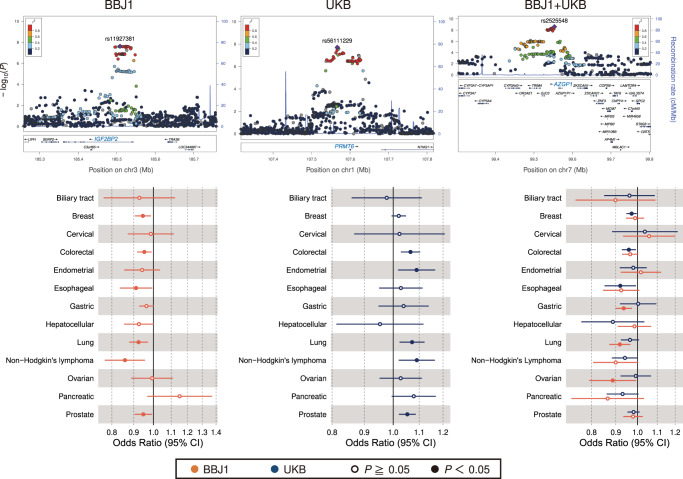
Novel loci identified in the all-cancer meta-analysis. Regional plots of the novel loci and forest plots of the lead loci variants identified in the all-cancer meta-analysis. Purple diamond symbols in the regional plots represent the lead variants of the loci. In the forest plots, dots indicate the odds ratios of the variant for each cancer and whiskers represent 95% confidence intervals. The number of cases and controls in each GWAS are shown in Table 1. All statistical tests are two-sided and not adjusted for multiple comparisons.

We then performed a pan-cancer GWAS meta-analysis (all-cancer meta-analysis) to detect common genetic factors underlying cancer etiology (Supplementary Data 2). In the meta-analysis within BBJ1 or UKB, we identified two novel loci (BBJ1: rs11927381 at *IGF2BP2* on 3q27, *P* = 2.4 × 10^−8^; UKB: rs56111229 at *PRMT6* on 1p13, *P* = 2.0 × 10^−8^; Table 2 and Supplementary Fig. 4a). In addition, the all-cancer meta-analysis across BBJ1 and UKB found a novel association (rs2525548 at *AZGP1* on 7q22, *P* = 2.5 × 10^−9^; Table 2 and Supplementary Fig. 4a). Our standard fixed-effects meta-analysis successfully detected the variants exhibiting general pleiotropy involving five or six cancers at *P* < 0.05 (Fig. 2). To account for correlations due to the overlapping subjects, we employed the Lin–Sullivan method^26^, which has been applied to the meta-analysis across multiple traits^27^. Quantile-quantile (QQ) plots and lambda values suggested no obvious bias from population stratification (Supplementary Fig. 4b). We also conducted a GWAS of any cancer in each cohort (=joint analysis) and then meta-analyzed across populations (Supplementary Fig. 7). While the joint analysis successfully detected the *PRMT6* locus in the UKB datasets and the *AZGP1* locus in the cross-population meta-analysis, the *IGF2BP2* locus did not satisfy the genome-wide threshold in the joint analysis of the BBJ1 datasets. The joint analysis did not identify any additional novel locus that the fixed-effects meta-analysis could not find. The lead variant obtained from the cross-population all-cancer meta-analysis was found in an intergenic region and nominally associated with gastric, lung, and ovarian cancer in BBJ1; and with breast, colorectal, and esophageal cancer in UKB (Fig. 2). The effect allele of rs2525548-G was suggested to have protective effects across cancers. We performed colocalization analysis with the eQTL data; variation in *TRIM4* gene expression across 11 cancer-related tissues was potentially responsible for the GWAS locus (SS *P*-value: 1.4 × 10^−5^−5.4 × 10^−5^, suggested threshold after multiple testing correction <0.002; Supplementary Fig. 6c). For rs56111229, the novel variant of the UKB all-cancer meta-analysis, our colocalization analysis also found that *PRMT6* was one of the putative target genes (Supplementary Fig. 6b). To validate the eQTL effects in East Asian datasets, we used the ImmuNexUT data^28^, which is a gene-regulation atlas of 28 immune cell types from the Japanese subjects. We confirmed the same effects on various immune cell types from East Asians for rs56111229 and rs2525548, which showed the eQTL effects in whole blood in the GTEx data (Supplementary Fig. 8).

For the replication analysis, we selected all the variants satisfying *P* < 1.0 × 10^−6^ from the discovery GWAS/meta-analysis including the BBJ1 datasets, and evaluated the associations between the variants and cancer risks using the BBJ2 datasets. First, we evaluated the two variants from the single cancer meta-analysis (rs2800691 and rs2076295 associated with breast and lung cancer, respectively) and the two variants from the all-cancer meta-analysis (rs11927381 and rs2525548). Of these, two variants (rs2076295 and rs11927381) were replicated with the nominal significance in the same directional effects (*P* < 0.05; Table 2). We then conducted the combined meta-analysis across the discovery and replication datasets. All four variants satisfied the genome-wide significance again, robustly supporting our findings (Table 2). In addition, the combined meta-analysis identified three additional cancer risk variants (rs77753011 at *RPH3A* on 12q24, *P* = 5.5 × 10^−15^; rs36079339 at *AIDA* on 1q41, *P* = 3.9 × 10^−10^; rs2059904 at *EDNRA* on 4q31, *P* = 1.2 × 10^−8^; Table 2), of these two were pleiotropic associations. Our comprehensive GWAS meta-analysis revealed novel pleiotropic associations enhancing our understanding of shared cancer susceptibility and showed the strengths of the pan-cancer and cross-population approaches.

### Pan-cancer heritability estimates and genetic correlations

**Fig. 3:**
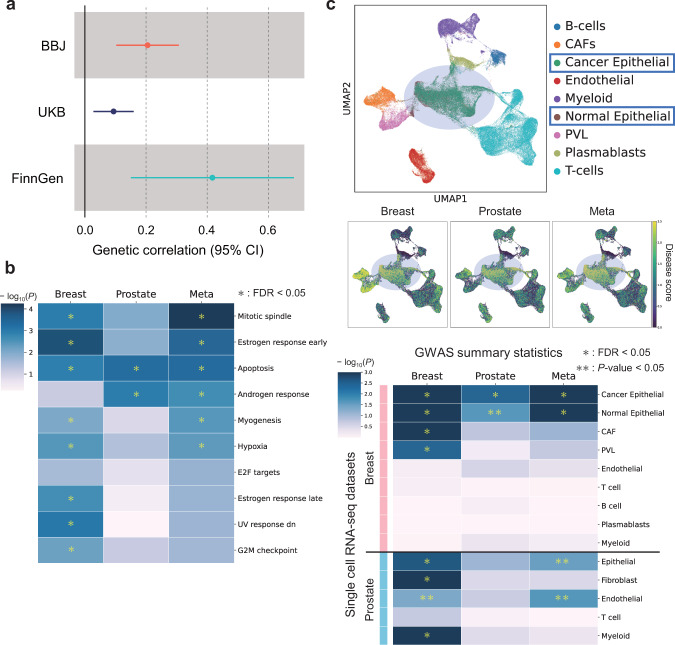
Breast and prostate cancer analysis. **a** Forest plot of genetic correlations between breast and prostate cancer in BBJ, UKB, and FinnGen. Dots indicate genetic correlations and whiskers represent 95% confidence intervals. **b** Heatmap describing the associations between the three GWAS meta-analyses of breast and prostate cancer and the top-ranking gene sets associated with the meta-analysis across breast and prostate cancer. The “Meta” column represents the meta-analysis across breast and prostate cancer. *P*-values of the heatmap are uncorrected and reflect two-sided tests. FDR was calculated via the Benjamini-Hochberg method across all gene sets. **c** Results of the cell type-specific analysis. UMAP visualizations of the breast cancer scRNA-seq dataset colored by cell type (top) and disease scores calculated via scDRS (middle). Heatmap describing the associations between the three GWAS meta-analyses of breast and prostate cancer and the cell types detected in the scRNA-seq datasets of breast and prostate cancer (down). The “Meta” column represents the meta-analysis across breast and prostate cancer. *P*-values of the heatmap are uncorrected and reflect two-sided tests. FDR was calculated via the Benjamini-Hochberg method across all cell types in each scRNA-seq dataset.

We evaluated the liability-scale heritability of each cancer from individual-level data using BOLT-REML^29^ (Table 1) and estimated genetic correlations among the 13 cancers (Supplementary Fig. 9). Among the 78 cancer pairs, 13 and four cancer pairs were genetically correlated in BBJ1 and UKB at the nominal significance level, respectively (Table 3). In BBJ1, three pairs were positively correlated after Bonferroni correction (*P* < 0.05/78 = 6.4 × 10^−4^): colorectal and gastric cancer (*r*_*g*_ = 0.30, *P* = 5.8 × 10^−6^), breast and lung cancer (*r*_*g*_ = 0.37, *P* = 2.1 × 10^−5^), and breast and prostate cancer (*r*_*g*_ = 0.21, *P* = 8.4 × 10^−5^). On the other hand, only one pair, colorectal and lung cancer, showed a significant correlation in UKB (*r*_*g*_ = 0.32, *P* = 2.3 × 10^−6^). While most of these relationships were observed in either population, only breast and prostate cancer consistently exhibited positive genetic correlations across East Asian and European ancestry. To validate this positive relationship, we applied linkage disequilibrium score regression (LDSC)^30^ to the GWAS summary statistics of the two cancers from FinnGen. Breast cancer was positively correlated with prostate cancer in Finnish populations as well (*r*_*g*_ = 0.42, *P* = 0.0021; Fig. 3a). Thus, we found consistent genetic correlations between breast and prostate cancer across populations.

**Table 3:** Genetic correlations (*r*_*g*_) calculated via BOLT-REML for the cancer pairs at *P* < 0.05

Cancer type 1	Cancer type 2	*r*_*g*_ (95% CI)	*P*
**BioBank Japan 1 (BBJ1)**			
Colorectal	Gastric	0.30 (0.17–0.43)	5.8 × 10^−6^
Breast	Lung	0.37 (0.20–0.54)	2.1 × 10^−5^
**Breast**	**Prostate**	**0.21 (0.10**–**0.31)**	**8.4** × **10**^**−5**^
Gastric	Lung	0.29 (0.11–0.46)	0.0014
Cervical	Lung	0.46 (0.16–0.76)	0.0030
Esophageal	Gastric	0.23 (0.06–0.39)	0.0069
Hepatocellular	Lung	0.38 (0.10–0.67)	0.0085
Gastric	Hepatocellular	0.30 (0.06–0.53)	0.013
Lung	Pancreatic	−0.47 (−0.90 to −0.05)	0.030
Colorectal	Pancreatic	0.36 (0.03–0.68)	0.032
Cervical	Esophageal	0.29 (0.02–0.55)	0.033
Endometrial	Non-Hodgkin’s lymphoma	0.57 (0.03–1.00)	0.037
Gastric	Non-Hodgkin’s lymphoma	0.27 (0.01–0.53)	0.046
**UK Biobank (UKB)**			
Colorectal	Lung	0.32 (0.19–0.45)	2.3 × 10^−6^
Breast	Endometrial	0.39 (0.15–0.62)	0.0014
**Breast**	**Prostate**	**0.094 (0.03**–**0.16)**	**0.0052**
Esophageal	Lung	0.23 (0.03–0.44)	0.025

*P* values are uncorrected. Genetic correlations between breast and prostate cancer are shown in bold.

*CI* confidence interval

### Breast and prostate cancer large-scale meta-analysis

We conducted a breast and prostate cancer large-scale meta-analysis, as we expected that the shared genetic backgrounds among the two cancers would boost the statistical power. In addition to the BBJ1, UKB, and FinnGen datasets, we utilized the summary statistics from the largest-to-date European ancestry GWAS of breast (BCAC)^31^ and prostate cancer (PRACTICAL; Supplementary Fig. 10a)^32^. We performed the large-scale GWAS meta-analysis within each cancer and the large-scale meta-analysis across breast and prostate cancer (Supplementary Fig. 10b and Supplementary Data 4 and 5). The breast cancer meta-analysis identified 183 loci that exceeded the genome-wide significance, including 29 loci newly satisfying the genome-wide significance. In prostate cancer, 177 significantly associated loci including 21 new associations were found. Of these, 11 breast cancer risk loci and one prostate cancer risk locus were previously reported with the suggestive significance (*P* < 1.0 × 10^−6^)^14,23^. Finally, we detected 218 genome-wide significant variants in the breast and prostate cancer meta-analysis. Of these, 8 variants were not previously associated with either breast or prostate cancer (Supplementary Data 6). Our replication meta-analysis incorporating the BBJ2 datasets showed that all the variants found from the large-scale meta-analysis met the genome-wide significance threshold again. In addition, we identified 33 additional novel loci associated with breast and/or prostate cancer from the replication meta-analysis (Supplementary Data 6). As expected, the shared heritability among the two cancers increased the statistical power; our large-scale meta-analysis could identify several novel associations, indicating genetic commonalities between breast and prostate cancer.

### Pathway enrichment analysis across breast and prostate cancer

For further biological insights, we performed the pathway enrichment analysis using PASCAL^33^ and assessed the associations between the three GWAS meta-analysis of breast and prostate cancer mentioned above and the hallmark gene sets from the Molecular Signature Database (MsigDB; Fig. 3b and Supplementary Data 7)^34^. Of these, six pathways including mitotic spindle, estrogen response early, apoptosis, androgen response, myogenesis and hypoxia were shared by both cancers and satisfied FDR < 0.05.

Next, we evaluated the functional characteristics of shared risk variants associated with both breast and prostate cancer at the nominal significance level (Supplementary Fig. 11). Our GWAS meta-analysis identified 218 lead variants associated with the two cancers (*P* < 5.0 × 10^−8^). Among them, 118 variants were nominally associated with both cancers (shared risk variants) and the rest were nominally associated with either cancer (cancer type-specific variants; Supplementary Fig. 11a). The 118 shared risk variants were relatively more enriched in intronic/exonic variants and active chromatin regions and held more eQTL variants compared with the 100 cancer type-specific variants (Supplementary Fig. 11b). The more stringent the threshold, the more apparent these characteristics were (Supplementary Fig. 11b). Furthermore, we conducted functional gene mapping using FUMA^35^ and the gene set enrichment analysis^36^ with the MsigDB hallmark gene sets (Supplementary Fig. 11a). While the 100 cancer type-specific variants were associated with only one gene set, the 118 shared risk variants were associated with 10 gene sets including G2M checkpoint, E2F targets, interferon alpha response, IL2-STAT5 signaling, estrogen response late, mTORC1 signaling, and cholesterol homeostasis (FDR < 0.05). We conducted the pathway analysis at the genome-wide and variant-specific levels and detected shared susceptibility pathways between breast and prostate cancer.

### Cell type-specific analysis of breast and prostate cancer

To acquire further insights into the common genetic basis between breast and prostate cancer, we conducted the cell type-specific analysis at single-cell resolution. Using scDRS^37^, we assessed cell type enrichment in single-cell RNA sequence (scRNA-seq) datasets of breast and prostate cancer^38,39^. First, we selected the top 2000 genes representing the polygenic cancer risk from the three large-scale meta-analysis mentioned above. We calculated a disease score for each cell in the scRNA-seq datasets using each of the three gene sets. We then evaluated the associations between the three gene sets and nine or five cell types in the scRNA-seq data of the two cancers, respectively. In the breast cancer scRNA-seq data, cells annotated as cancer/normal epithelial showed higher disease scores than other cell types across all gene sets; all gene sets were associated with cancer/normal epithelial cells (Fig. 3c and Supplementary Data 8). For some known genes related to breast cancer (e.g., *MYC*, *IGFBP5*, *CCND1*, *ESR1*), we confirmed epithelial cells showed higher expression levels (Supplementary Fig. 12). We observed similar associations in the prostate cancer scRNA-seq data (Fig. 3c). In addition, the breast cancer gene set was uniquely associated with cancer-associated fibroblasts (CAF) and perivascular-like cells (PVL) in the breast cancer scRNA-seq data. Our cell type-specific analysis at a single-cell level demonstrated distinct and common genetic components among breast and prostate cancer.

## Discussion

In this study, we performed the pan-cancer and cross-population GWAS meta-analysis on the 13 cancers and identified ten loci newly satisfying the genome-wide significance. The single cancer GWAS/meta-analysis found five risk variants of the specific individual cancers across East Asians and Europeans (e.g., *PADI6* on 1p36 and *DSP* on 6p24). On the other hand, five pleiotropic loci were detected in the all-cancer meta-analysis (e.g., *IGF2BP2* on 3q27, *PRMT6* on 1p13, and *TRIM4* on 7q22). With increasing use of biobank resources, the time-to-event data analysis is becoming informative to detect genetic variants predicting the disease prognosis. Using SPACox^40^ and the survival data in BBJ, we investigated the survival impacts of the variants identified in the single/all cancer GWAS/meta-analysis. We did not find any significant associations between the cancer risk variants and survival (Supplementary Data 9). We then found positive genetic correlations between breast and prostate cancer across populations. Further, our large-scale meta-analysis of breast and prostate cancer identified 91 loci newly satisfying the genome-wide significance threshold. The downstream analysis showed common pathways including apoptosis and the associations between the polygenic risk of both cancers and epithelial cells.

The novel risk variant of lung cancer is an intronic *DSP* variant previously associated with interstitial lung disease^41^ or lung function^42^. DSP is a structural component of desmosomes^43^. In human non-small cell lung cancer, DSP has been reported to function as a tumor suppressor through inhibition of the Wnt/β-catenin signaling pathway^44^. In addition, in the all-cancer meta-analysis, we identified a novel variant near *TRIM4*. TRIM family proteins are involved in various cellular processes such as cell cycle and apoptosis^45^; particularly, TRIM4 has been reported to sensitize cells to oxidative stress induced cell death^46^. GWAS have offered genetic evidence highlighting targets of drug discovery and repositioning^8^. Our all-cancer meta-analysis within UKB detected an association within the *PRMT6* region. PRMT6 is involved in epigenetic regulation through methylation and plays different roles in various cancers^47^. The study of PRMT6 inhibitors has been increasing to explore their efficacy as potential cancer therapy for various cancers^48^. Especially for the pleiotropic loci from our study, the downstream analysis investigating the functional roles of the putative target genes across multiple cancers is warranted. We anticipate that further research based on our findings can become a steppingstone to anticancer therapy across cancers and contribute to a better understanding of common cancer susceptibility.

In our analysis of shared heritability among the cancers, several cancer pairs in the UKB datasets showed similar genetic correlations with the previous reports: esophagus/stomach and lung cancer (*r*_*g*_ = 0.44, *P* = 0.0035)^14^ and colorectal and lung cancer (*r*_*g*_ = 0.28, *P* = 6.6 × 10^−7^)^13^. We confirmed the positive genetic correlations between breast and prostate cancer across populations. A previous study showed a nominal genome-wide correlation (*r*_*g*_ = 0.07, *P* = 0.012) and significant local correlations (9p21: *r*_*g*_ = 4.6 × 10^−4^, *P* = 1.0 × 10^−6^; 10q26: *r*_*g*_ = −9.8 × 10^−4^, *P* = 1.0 × 10^−7^) between the two cancers using the summary statistics of BCAC and PRACTICAL^13^. Another epidemiological study reported that a prostate cancer family history in a first-degree relative was associated with a higher incidence rate of breast cancer and vice versa^49^. Considering these findings, the two cancers appear to share substantial heritable risk.

The large-scale GWAS meta-analysis across the two cancers identified 15 novel loci. Of these, ten were previously associated with sex hormone-binding globulin (SHBG) and/or testosterone levels^50^. Recent Mendelian randomization (MR) studies indicated potential causal inference of sexual hormone traits on the risk of breast and prostate cancer^50,51^. The MR analysis by Ruth et al. revealed potential causal effects of sex hormones on hormone-sensitive cancers and indicated that higher bioavailable testosterone levels increased the risk of prostate cancer and estrogen receptor-positive breast cancer^50^. Our GWAS findings supported the impacts of testosterone on both cancers’ susceptibility and suggested that sexual hormone traits such as SHBG or testosterone levels may be a key mediator of shared mechanisms between the two cancers. Our pathway enrichment analysis also suggested that pathways related to sexual hormone responses have a significant effect in breast and prostate cancer susceptibility. Last, we performed the cell type-specific analysis at a single-cell level. The transcriptomic examination of individual cells overcame the limitations of bulk analysis and enabled a more refined dissection of the cellular basis of cancer^52^. Our results indicated shared heritability between the two cancers was enriched in epithelial cells. In breast cancer, germline factors were suggested to affect the tumor microenvironment including mesenchymal cells. At single-cell resolution, our cell type-specific analysis showed unique and shared genetic basis across breast and prostate cancer. In the cross-cancer approach, focusing on the genetically correlated cancers showed novel potential to reveal common genetic components underlying carcinogenesis, which might be shared across multiple cancers. We consider that future research including larger sample sizes from diverse populations would detect additional correlated cancer pairs. By focusing on such cancer pairs, de novo cancer grouping can contribute to understanding human cancer genetics.

Several limitations need to be acknowledged in this study. Our analysis covered a wide range of 13 cancers, but we did not include less common ones due to data unavailability. To maximize the power, we leveraged two biobank resources. However, the sample size of some cancers was limited. Future studies should focus on larger sample sizes and include rare cancers. The differences of genotyping platforms and imputation procedures between the cohorts can be potential limitations. We consider that future studies using various pipelines would be helpful to validate our findings and accumulate our knowledge of cancer genetics. Since we focused on common variants in this study, the contribution of rare variants or the interactions between germline and somatic mutations requires further research.

In summary, our comprehensive and large-scale genomic study revealed novel pleiotropic associations and highlighted the advantages of the pan-cancer analysis. Using the biobank resources from East Asians and Europeans, our study contributed to deciphering the global landscape of heritable cancer risk. Furthermore, our results demonstrate that focusing on the genetically correlated cancers is a promising approach to understand the shared genetic backgrounds underlying carcinogenesis.

## Methods

### Subjects

In the discovery GWAS/meta-analysis, all the Japanese subjects were included in BBJ1, a prospective biobank that collaboratively recruited approximately 200,000 patients with ≥1 of 47 diseases and collected DNA, serum samples, and clinical information from 12 medical institutions in Japan between 2003 and 2007^15,53^. The samples for the replication analysis were registered in BBJ2 between 2013 and 2018, which included ~80,000 new patients with 38 target diseases. Among them, the cases diagnosed with any of the following 13 cancers (biliary tract, breast, cervical, colorectal, endometrial, esophageal, gastric, hepatocellular, lung, non-Hodgkin’s lymphoma, ovarian, pancreatic, and prostate cancer) were analyzed. As controls, we used subjects without cancer. All the participants in BBJ provided written, informed consent approved by ethics committees of the Institute of Medical Sciences, the University of Tokyo and RIKEN Center for Integrative Medical Sciences. For the European subjects, we extracted cancer cases and controls from UKB, a population-based cohort of ~500,000 people aged 40–69 across the United Kingdom^54^. We included the incident cases in UKB, where the last follow-up date was 2021/4/16. This study was approved by the ethical committee of Osaka University Graduate School of Medicine.

### Genotyping and imputation

We genotyped the Japanese samples in BBJ1 with the Illumina HumanOmniExpressExome BeadChip or a combination of the Illumina HumanOmniExpress and HumanExome BeadChips. Quality control of samples and genotypes was conducted as described elsewhere^55^. We analyzed subjects of East Asian ancestry identified by principal component analysis (PCA)-based criteria. Genotype data were imputed with 1000 Genomes Project Phase 3 (version 5) genotype data and Japanese whole-genome sequencing data using Minimac3^21^. We genotyped the BBJ2 subjects for replication using Illumina Asian Screening Array chip and excluded individuals with a low call rate (<0.98) and outliers from the PCA-based East Asian cluster. Quality control of the variants was conducted using the following criteria: (i) with a low call rate (<0.99); (ii) with low minor allele counts (<5); and (iii) with Hardy–Weinberg equilibrium test *P* value < 1.0 × 10^−10^. We imputed dosages with the combined reference panel of 1000 Genomes Project Phase 3 and Japanese whole-genome sequencing data using Minimac4. Subjects in UKB were genotyped using the Applied Biosystems UK BiLEVE Axiom Array or the Applied Biosystems UK Biobank Axiom Array. After quality control, genotype data were imputed with the Haplotype Reference Consortium data and the merged UK10K and 1000 Genomes Project Phase 3 reference panels using IMPUTE4^16^. We analyzed Caucasian subjects identified by PCA-based criteria.

### Single cancer GWAS

We conducted single cancer GWAS for each cancer in a single ancestry using a generalized linear mixed model (GLMM) implemented in SAIGE (v0.43)^56^ to adjust for case-control imbalance. We included age, sex, and the top five principal components as covariates and used the leave-one-chromosome-out scheme to avoid proximal contamination. We excluded variants with an imputation quality Rsq <0.7 or minor allele frequency <0.01. We set the genome-wide significant threshold at *P* < 5.0 × 10^−8^. We considered the HLA region (chromosome 6: 26–34 Mbp) as one locus due to its complex and strong linkage disequilibrium (LD) structure^57^.

### GWAS meta-analysis

We applied a standard fixed-effects approach implemented in RE2C^58^ for GWAS meta-analysis. For the all-cancer meta-analysis, we employed the Lin-Sullivan method^26^ to account for correlations from overlapping samples. This strategy has been applied to meta-analysis across multiple traits^27^. We defined a locus as a genomic region within ±500 Kbp from the lead variant and considered a locus as novel if the lead variant and its nearest gene were not previously associated with the cancers of interest (*P* < 5.0 × 10^−8^) by querying the GWAS Catalog^18^, PhenoScanner V2^19^, PheWeb^20^, PheWeb.jp^21^, and Open Targets Genetics^22^. To help ensure that the identified loci were novel, we confirmed that any variants within ±1 Mbp from the lead variants and correlated with the lead variants (*r* ^2^ > 0.1) were not previously reported at *P* < 1.0 × 10^−6^ using GWAS Catalog. For the locus previously reported with the suggestive significance (*P* < 1.0 × 10^−6^), we used the descriptions as newly satisfying the genome-wide significance. Regional plots of novel loci were created using LocusZoom^59^. We obtained the functional annotations of lead variants using ANNOVAR^60^ and Open Targets Genetics.

### Colocalization analysis

We used eQTL data from the GTEx v8 database^24^ and performed colocalization analysis with LocusFocus (v1.4.9)^25^. The Simple Sum method implemented in LocusFocus is a frequentist colocalization method to identify the most relevant genes and tissues for a particular GWAS locus in the presence of high LD and allelic heterogeneity. We selected 1000 Genomes Project European populations to calculate the LD matrix and obtained SS *P*-values for gene-tissue pairs. We used a Bonferroni-corrected threshold for significant colocalization when assessing ≥2 gene-tissue pairs.

### Heritability and genetic correlation

We estimated the heritability of each cancer explained by the genotyped SNPs (single nucleotide polymorphisms) and genetic correlations among cancer pairs using BOLT-REML (v2.3.6)^29^. BOLT-REML applies variance component analysis and a Monte Carlo algorithm. We included age, sex, and the top five principal components as covariates. We reported the heritability on the liability scale adjusted for lifetime risks of each cancer based on SEER 2015–2017 estimates^61^. For FinnGen datasets, we applied LDSC (v1.0.1)^30^ with the 1000 Genomes Project European reference panel to estimate the genetic correlation between breast and prostate cancer. LDSC used HapMap3 SNPs and the analysis did not include population specific variants like founder mutations of *BRCA2* or *CHEK2* in the Finnish population.

### Pathway enrichment analysis

We conducted the pathway enrichment analysis at the genome-wide level using PASCAL^33^ and evaluated the associations between the summary statistics of the breast and prostate cancer large-scale meta-analysis and the MsigDB (v7.5.1) hallmark gene sets. PASCAL uses 1000 Genomes Project European data to correct for LD structure and computed pathway enrichment scores. We used empirical scores and set the threshold for significant pathway enrichment at FDR < 0.05. For the functional characterization of the 218 lead variants found in large-scale GWAS meta-analysis across breast and prostate cancer, we used FUMA (v1.3.8)^35^ to obtain functional, eQTL, and chromatin feature annotations. We defined an eQTL variant one with ≥1 significant eQTL effect in the GTEx database and an active chromatin region as the most common state value ≤7 according to Roadmap’s 15-core chromatin states across 127 cell or tissue types^62^. For shared risk variants and cancer type-specific variants, we conducted functional gene mapping using FUMA and gene set enrichment analysis (v4.2.3)^36^ using the MsigDB hallmark gene sets as in a previous study^14^.

### Cell type-specific analysis

Using scDRS (v1.0.0)^37^, we assessed the cell type enrichment in the scRNA-seq datasets of breast and prostate cancer. First, we used MAGMA (v1.10)^63^ to calculate the gene *P*-value and Z-score from the summary statistics of the breast and prostate cancer large-scale meta-analysis and selected the top 2000 genes as a set of putative disease genes. Second, scDRS calculated a disease score of each cell in the scRNA datasets by aggregating the expression of the putative disease genes and computed a set of 1,000 Monte Carlo control scores using a random gene set. Third, scDRS normalized these scores and calculated a *P*-value for individual cells. We used the compute_score function with default parameters and included the number of genes per cell and clinical subtypes in the breast cancer scRNA-seq dataset as covariates. Finally, scDRS performed the cell type-level analysis to associate the putative gene sets with the cell types in the scRNA-seq datasets using the compute_downstream function with default settings.

### Survival analysis

We used SPACox^40^ and the survival data in BBJ to assess the association between survival and the variants found in the single/all cancer GWAS/meta-analysis including the East Asian datasets. We investigated the impact of the variants on all-cause death among the samples with each cancer. The COX proportional hazards model was adjusted for age, sex, and the top five principal components. We considered a variant satisfying *P* < 0.0038 (0.05/13) as statistically significant.

### Reporting summary

Further information on research design is available in the Nature Portfolio Reporting Summary linked to this article.

## Supplementary information

Supplementary Information

Description of Additional Supplementary Files

Supplementary Data 1-9

Reporting Summary

## Data Availability

GWAS genotype data of the BBJ are available at the NBDC Human Database (research ID: hum0014 and hum0311). All the GWAS summary statistics of our study are publicly available at the NBDC Human Database (research ID: hum0197) and PheWeb.jp (https://pheweb.jp/) without restriction. The UKB analysis was conducted via application number 47821 (https://www.ukbiobank.ac.uk/). We used the FinnGen release 6 data. Summary results can be accessed through application at https://www.finngen.fi/en/access_results/. The summary statistics of BCAC and PRACTICAL are available at http://bcac.ccge.medschl.cam.ac.uk/ and http://practical.icr.ac.uk/blog/, respectively. The breast cancer scRNA-seq data are available for download through the Broad Institute Single Cell portal at https://singlecell.broadinstitute.org/single_cell/study/SCP1039. The prostate cancer scRNA-seq data have been deposited in the Gene Expression Omnibus (GEO) under accession no. GSE141445 and the Genome Sequence Archive for Human (GSA-Human) under accession HRA000312 and can be accessed at http://www.pradcellatlas.com/. The GTEx v8 and ImmuNexUT data were obtained from the GTEx portal (https://gtexportal.org/home/) and the ImmuNexUT website (https://www.immunexut.org/), respectively.

## Code availability

We used the publicly available software for the analysis. The software used is described in the Methods section.
