# Supplementary material for: Pan-cancer and cross-population genome-wide association studies dissect shared genetic backgrounds underlying carcinogenesis
Source: Nat Commun. 2023 Jun 20;14:3671. doi: 10.1038/s41467-023-39136-7 (PMC10282036; doi:10.1038/s41467-023-39136-7)
Supplement: Supplementary file 1 — Supplementary Information [file 41467_2023_39136_MOESM1_ESM.docx]

**Pan-cancer and cross-population genome-wide association studies dissect shared genetic backgrounds underlying carcinogenesis.**

Go Sato et al.

Corresponding to Prof. Yukinori Okada (yokada@sg.med.osaka-u.ac.jp).


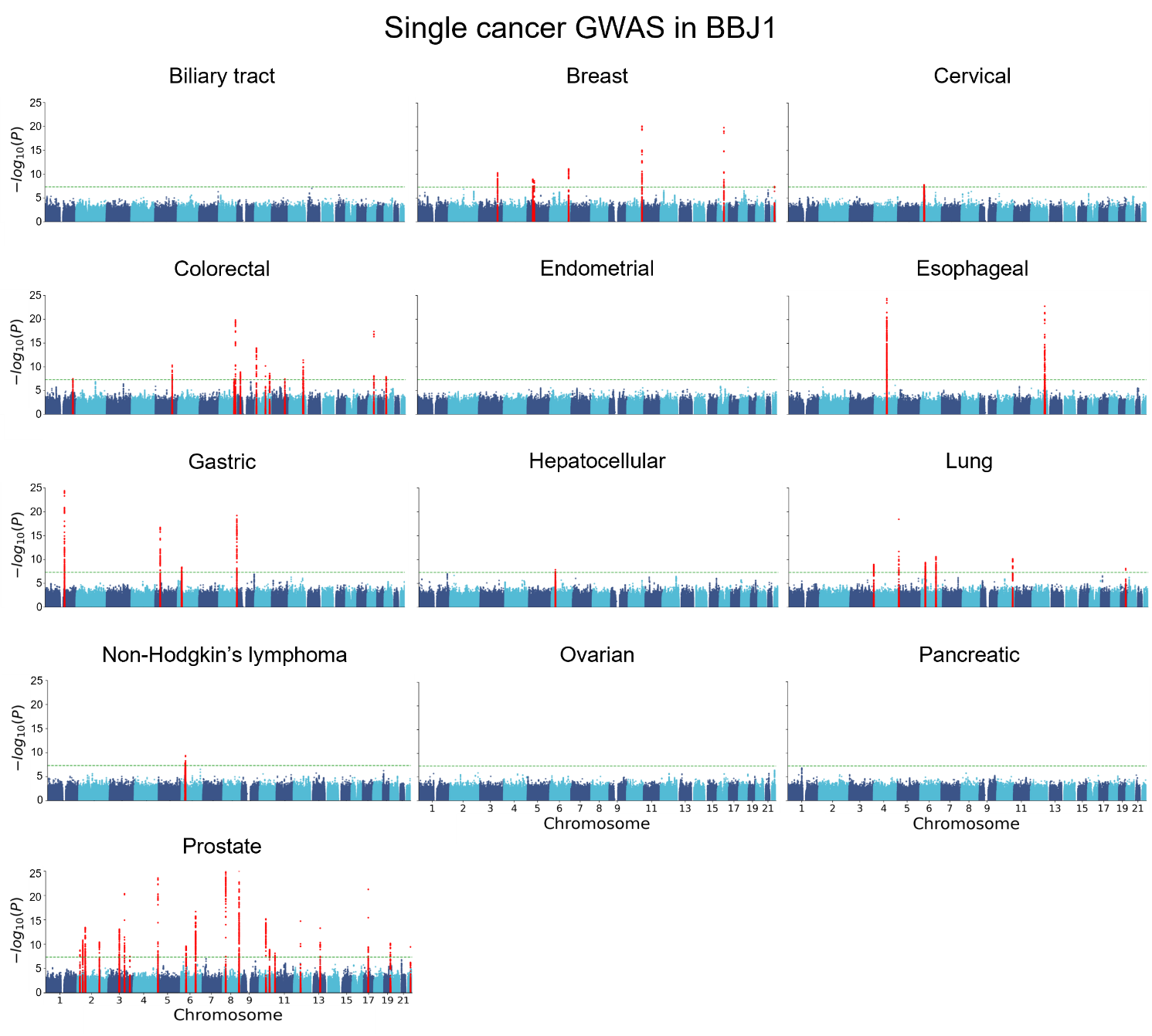
**Supplementary Fig. 1. Manhattan plots of the single cancer GWAS in BBJ1.**

Manhattan plots of the single cancer GWAS in BBJ1. The genome-wide significant loci are colored in red. The upper limit was set to 25. The horizontal dashed green line represents the genome-wide significance threshold (*P* = 5.0 × 10^-8^). All statistical tests are two-sided and not adjusted for multiple comparisons.


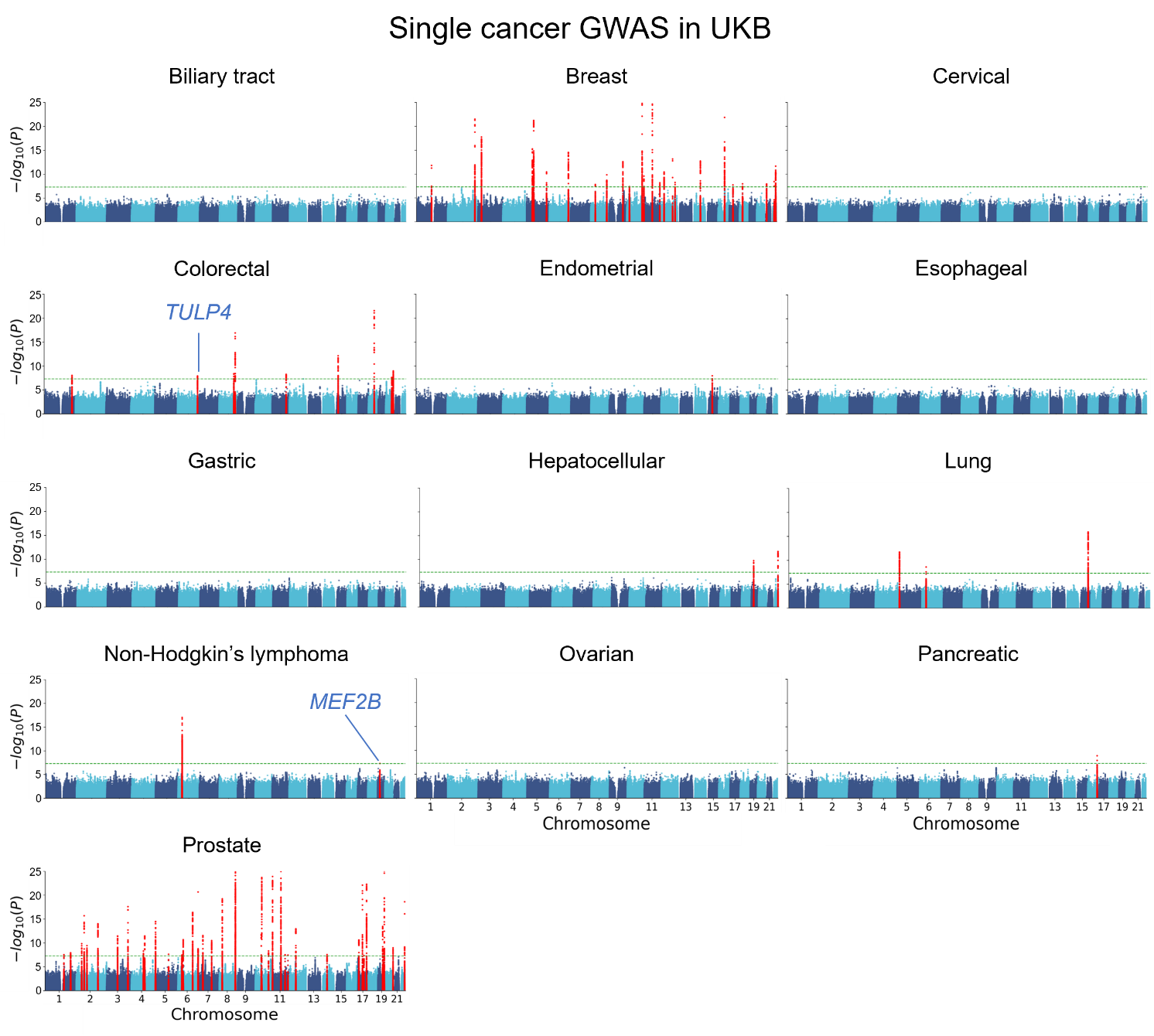
**Supplementary Fig. 2. Manhattan plots of the single cancer GWAS in UKB.**

Manhattan plots of the single cancer GWAS in UKB. Loci in red are genome-wide significant and loci indicated in blue are the novel ones identified in our study. The upper limit was set to 25. The horizontal dashed green line represents the genome-wide significance threshold (*P* = 5.0 × 10^-8^). All statistical tests are two-sided and not adjusted for multiple comparisons.


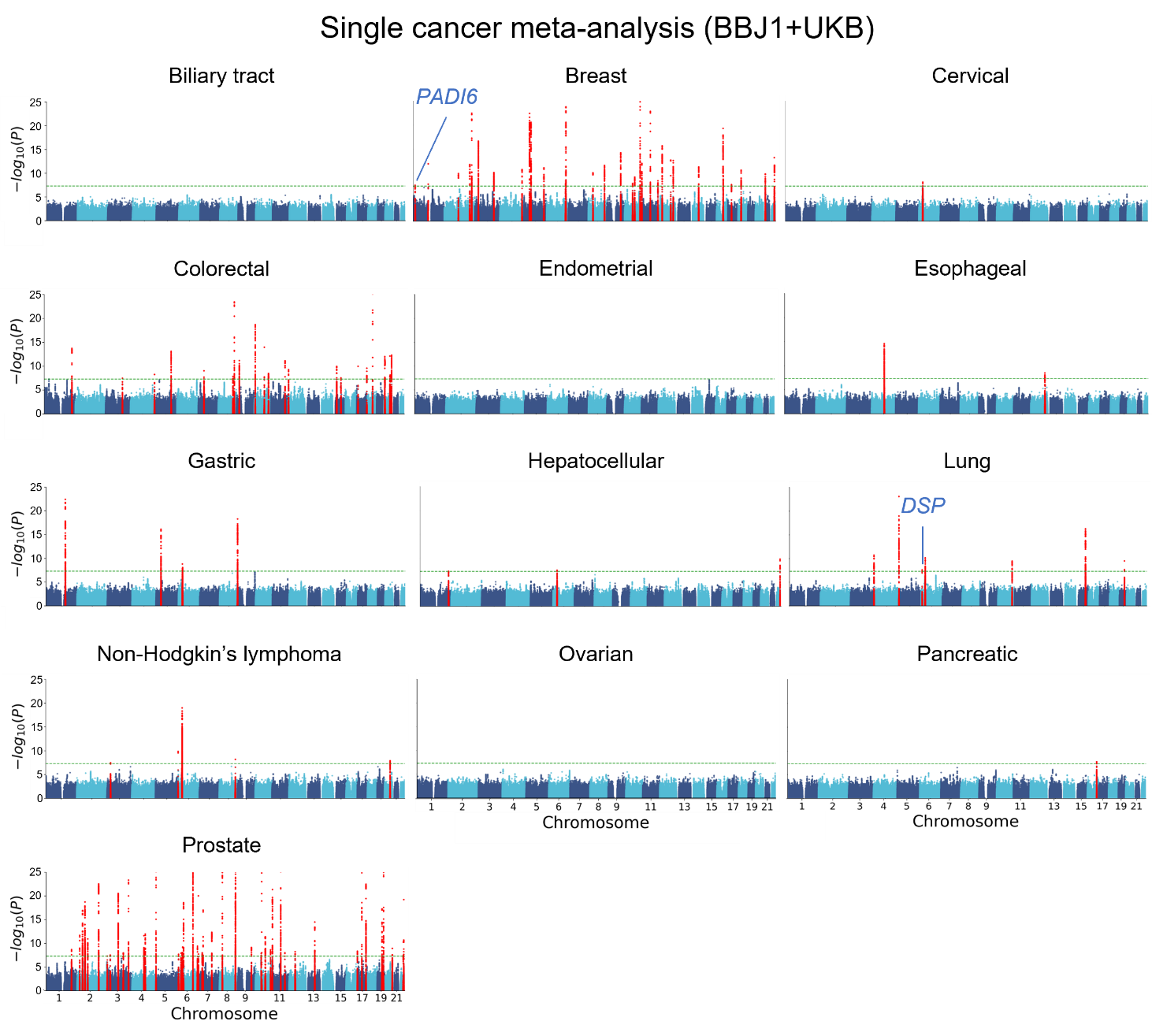
**Supplementary Fig. 3. Manhattan plots of the single cancer meta-analysis (BBJ1+UKB).**

Manhattan plots of the cross-population GWAS meta-analysis for each cancer (single cancer meta-analysis). The genome-wide significant loci are colored in red. The upper limit was set to 25. Loci newly satisfying the genome-wide significance are colored in blue. The horizontal dashed green line represents the genome-wide significance threshold (*P* = 5.0 × 10^-8^). All statistical tests are two-sided and not adjusted for multiple comparisons.


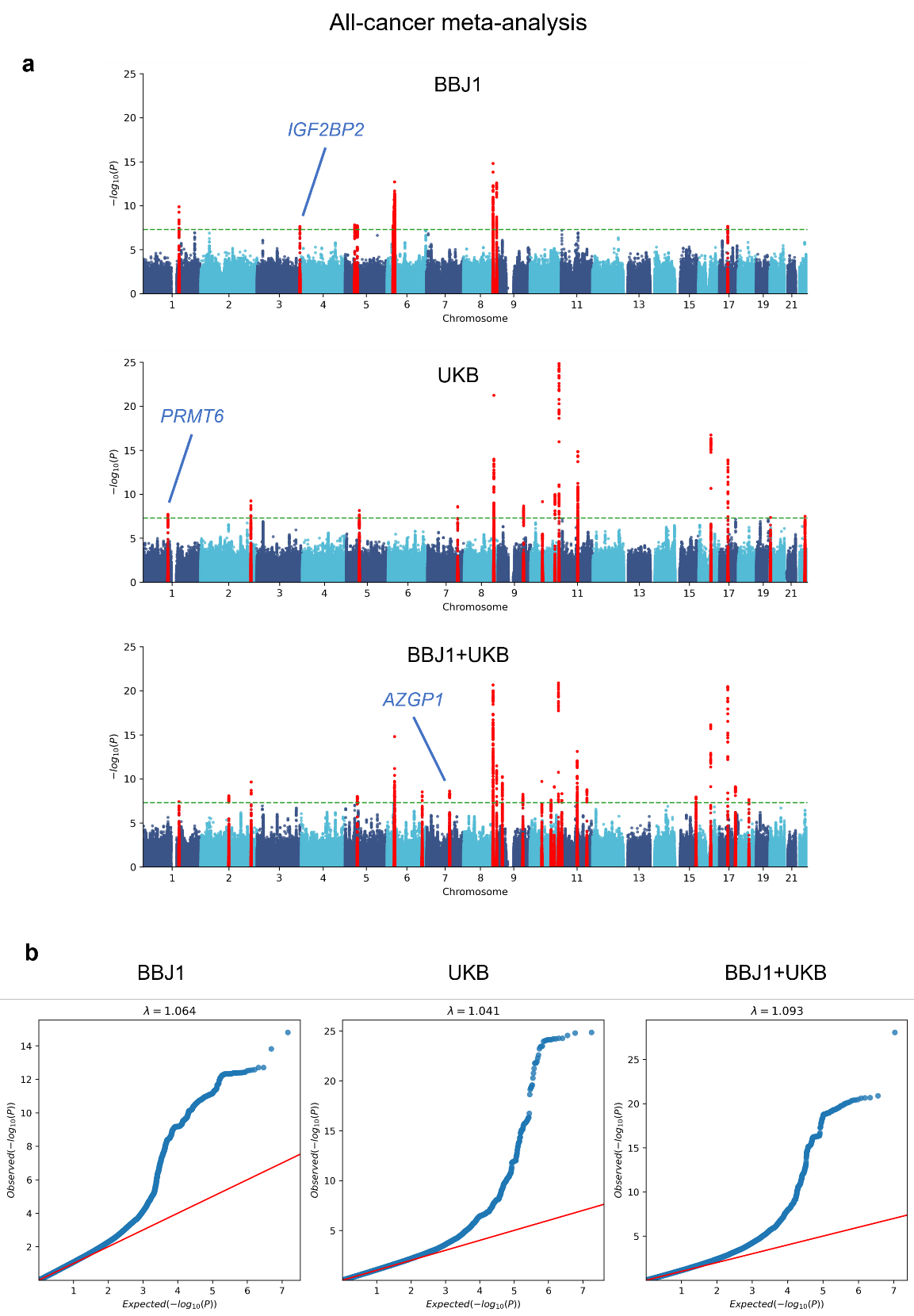
**Supplementary Fig. 4. Manhattan plots and QQ plots of the all-cancer meta-analysis.**

**a** Manhattan plots of the pan-cancer GWAS meta-analysis (all-cancer meta-analysis). Loci in red are genome-wide significant and loci indicated in blue are the novel ones identified in our study. The upper limit was set to 25. The horizontal dashed green line represents the genome-wide significance threshold (*P* = 5.0 × 10^-8^). All statistical tests are two-sided and not adjusted for multiple comparisons. **b** Quantile–quantile (QQ) plots of the all-cancer meta-analysis. Genomic inflation factors (λ_GC_) of the individual meta-analysis are shown above the figures.


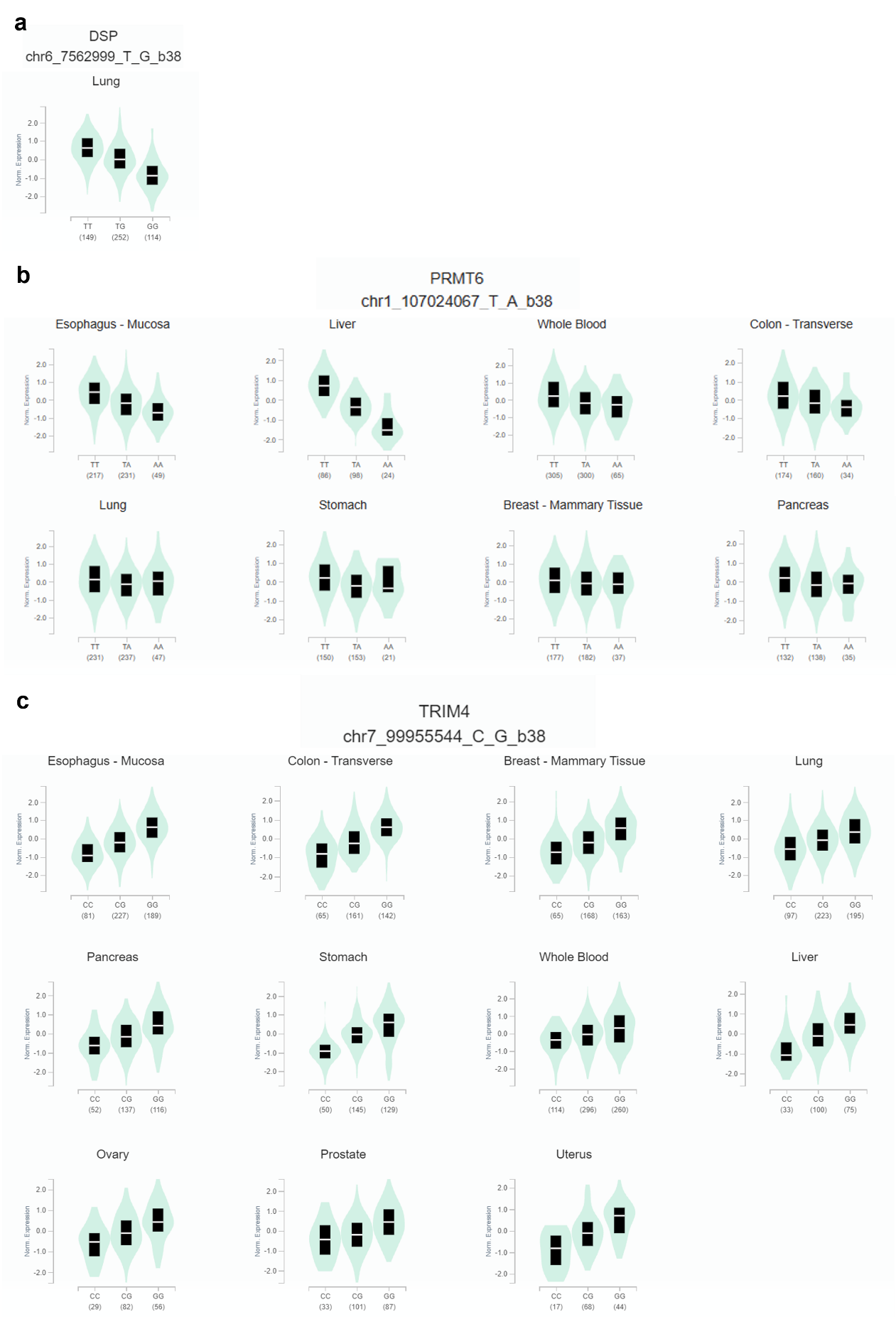
**Supplementary Fig. 5. The eQTL effects of the novel variants in the GTEx database.**

**a** Box plot of *DSP* expression levels in lung for rs2076295. **b** Box plots of *PRMT6* expression levels in eight cancer-related tissues for rs56111229. **c** Box plots of *TRIM4* expression levels in 11 cancer-related tissues for rs2525548. The numbers of the samples are shown below each genotype. White lines and box bounds indicate the median and the interquartile range, respectively.


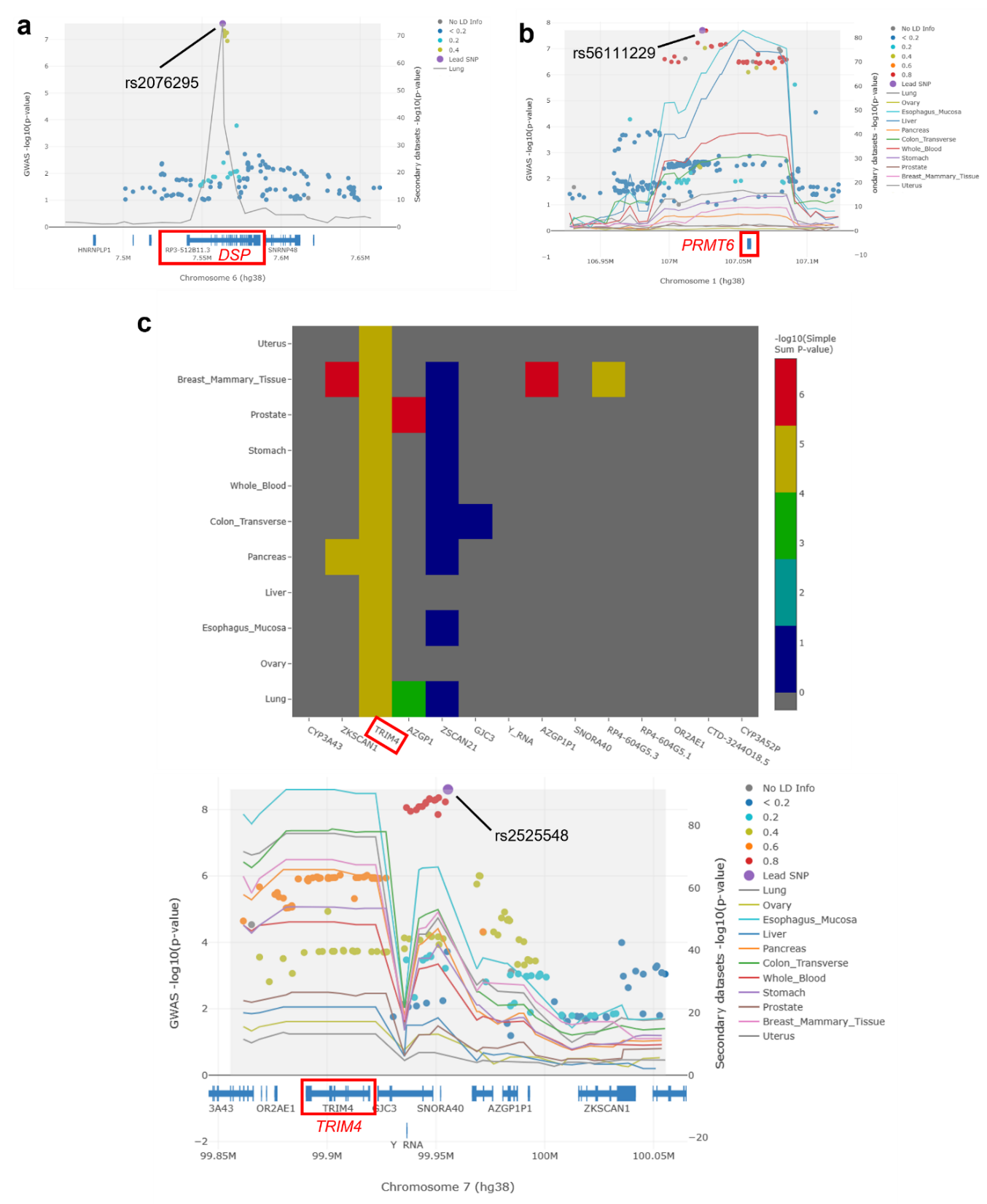
**Supplementary Fig. 6. Colocalization analysis in the novel loci.**

**a** Regional plot for the colocalization analysis in *DSP* locus. The dots indicate the -log_10_(*P*-value) in the GWAS meta-analysis and the solid line represents the -log_10_(*P*-value) in the eQTL data of *DSP* in lung. **b** The colocalization analysis in the novel locus identified in the all-cancer meta-analysis within UKB. In the regional plot, the dots indicate the -log_10_(*P*-value) in the GWAS meta-analysis and the solid lines represent the -log_10_(*P*-value) in the eQTL data of *PRMT6* in 11 cancer-related tissues. **c** The colocalization analysis in the novel locus identified in the all-cancer meta-analysis across BBJ1 and UKB. Heatmap describing the associations between 11 cancer-related tissues and 14 genes in the locus (upper). Regional plot in the locus (lower). The dots indicate the -log_10_(*P*-value) in the GWAS meta-analysis and the solid lines represent the -log_10_(*P*-value) in the eQTL data of *TRIM4* in the 11 tissues. *P*-values are uncorrected and reflect two-sided tests.


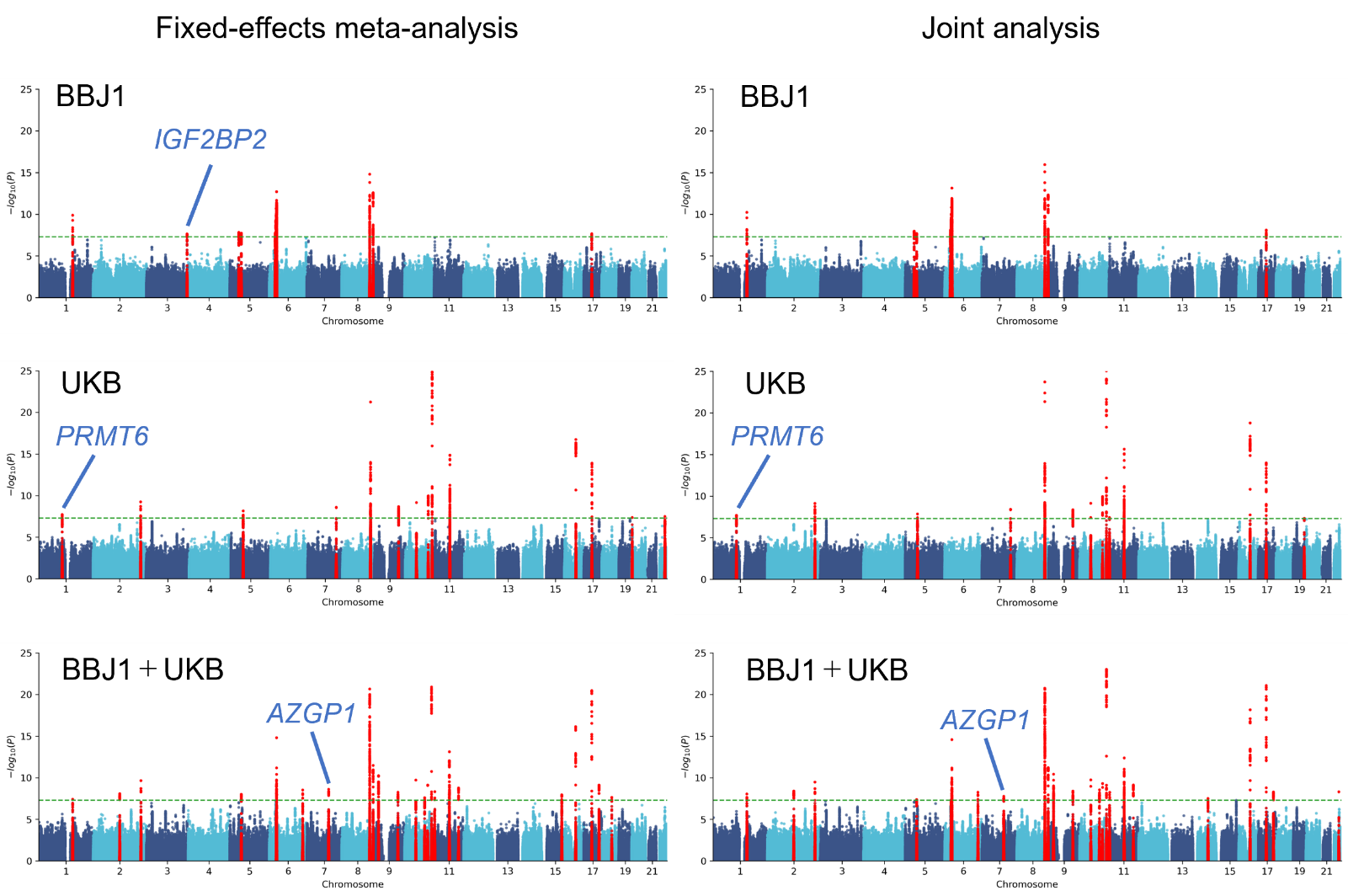
**Supplementary Fig. 7. Comparison between the fixed-effects meta-analysis and joint analysis.**

Manhattan plots of the fixed-effects meta-analysis (all-cancer meta-analysis) and joint analysis. Loci in red are genome-wide significant and loci indicated in blue are the novel ones identified in our study. The upper limit was set to 25. The horizontal dashed green line represents the genome-wide significance threshold (*P* = 5.0 × 10^-8^). All statistical tests are two-sided and not adjusted for multiple comparisons.


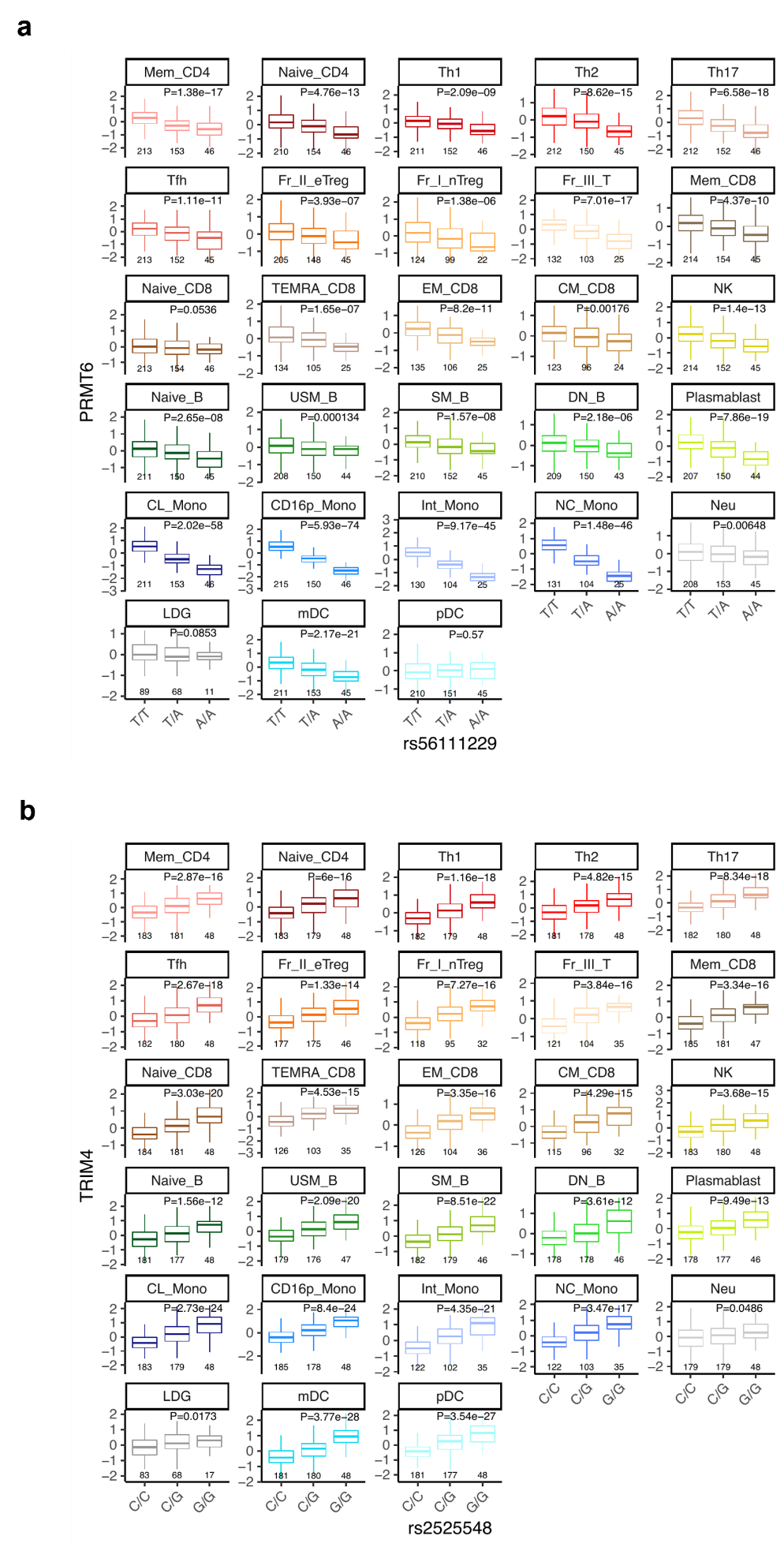
**Supplementary Fig. 8. The eQTL effects of the novel variants in the ImmuNexUT database.**

**a** Box plots of *PRMT6* expression levels in 28 immune cell types for rs56111229. **b** Box plots of *TRIM4* expression levels in 28 immune cell types for rs2525548. The numbers of the samples are shown below each box. Center lines and box bounds indicate the median and the interquartile range, respectively. *P*-values are uncorrected and reflect two-sided tests.


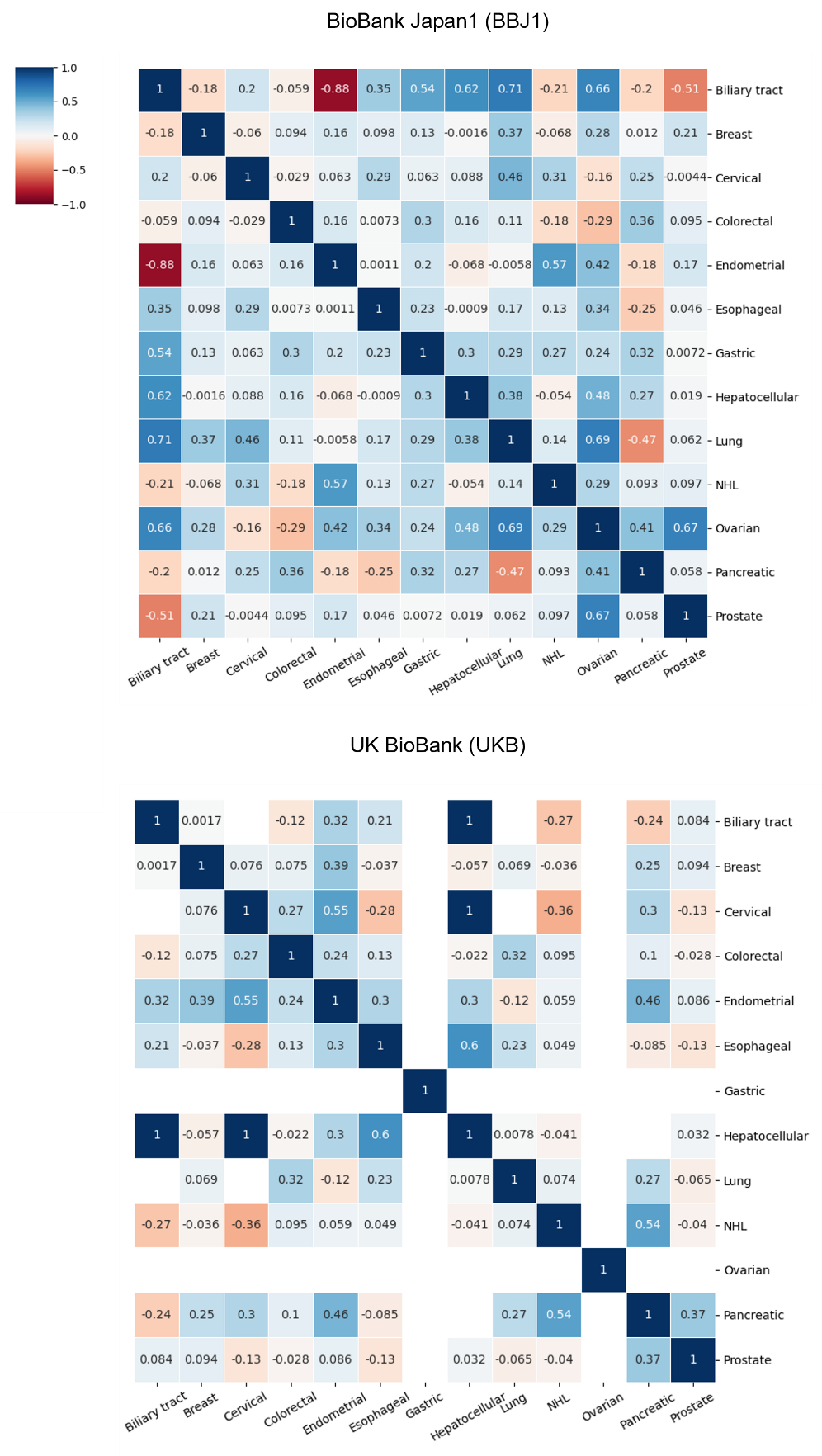
**Supplementary Fig. 9. Pan-cancer genetic correlations.**

Heatmaps describing genetic correlations of the 78 cancer pairs in the BBJ1 and UKB datasets. In the UKB datasets, some pairs including the cancers with low sample sizes or low heritability showed extremely high or low values and the estimates of these pairs were masked with white. NHL; non-Hodgkin’s lymphoma.


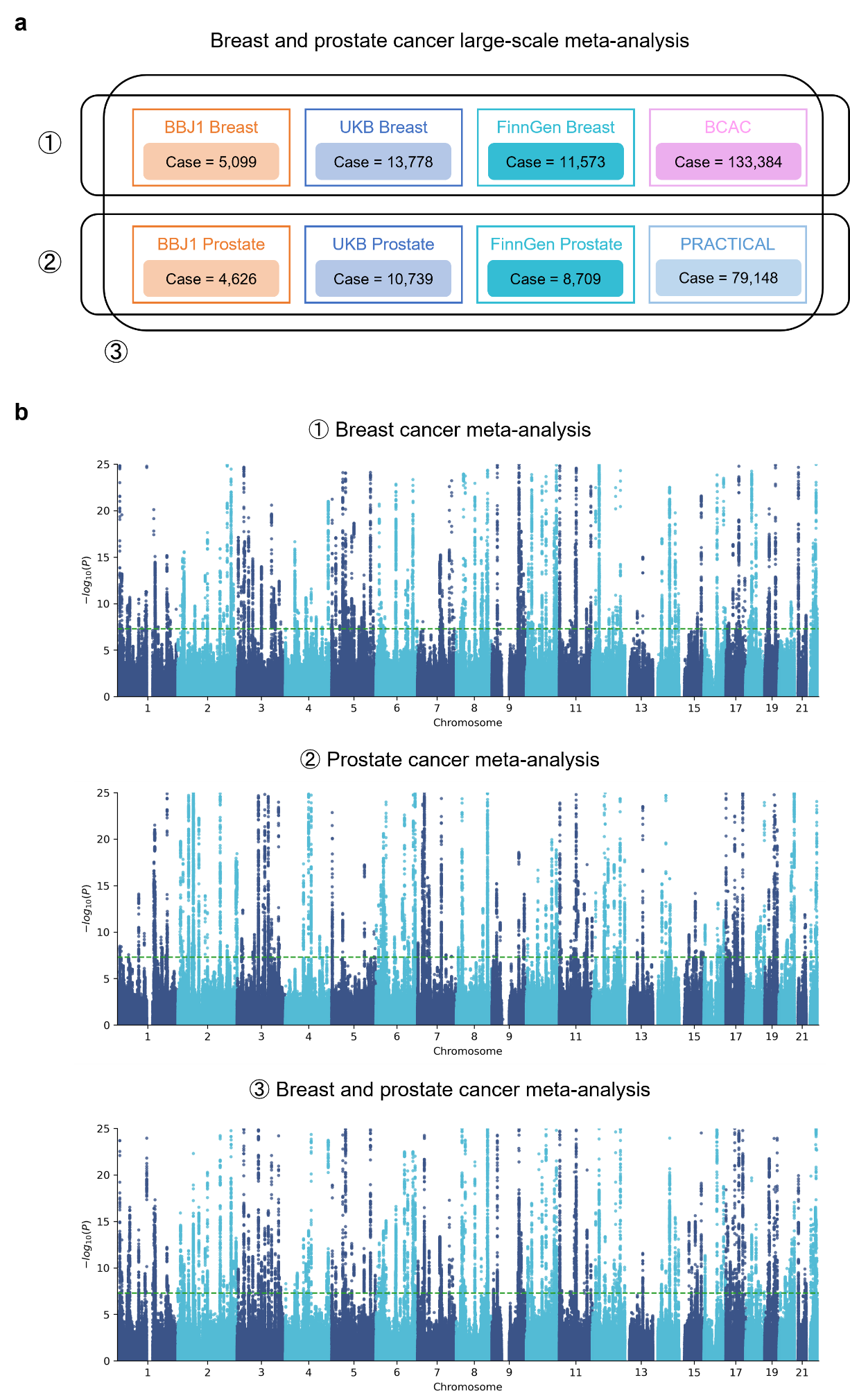
**Supplementary Fig. 10. Breast and prostate cancer large-scale meta-analysis.**

**a** Overview of the breast and prostate cancer large-scale meta-analysis integrating 8 GWAS datasets. **b** Manhattan plots of the breast and prostate cancer large-scale meta-analysis. The dashed green line indicates the genome-wide significance threshold (*P* = 5.0 × 10^-8^). All statistical tests are two-sided and not adjusted for multiple comparisons.


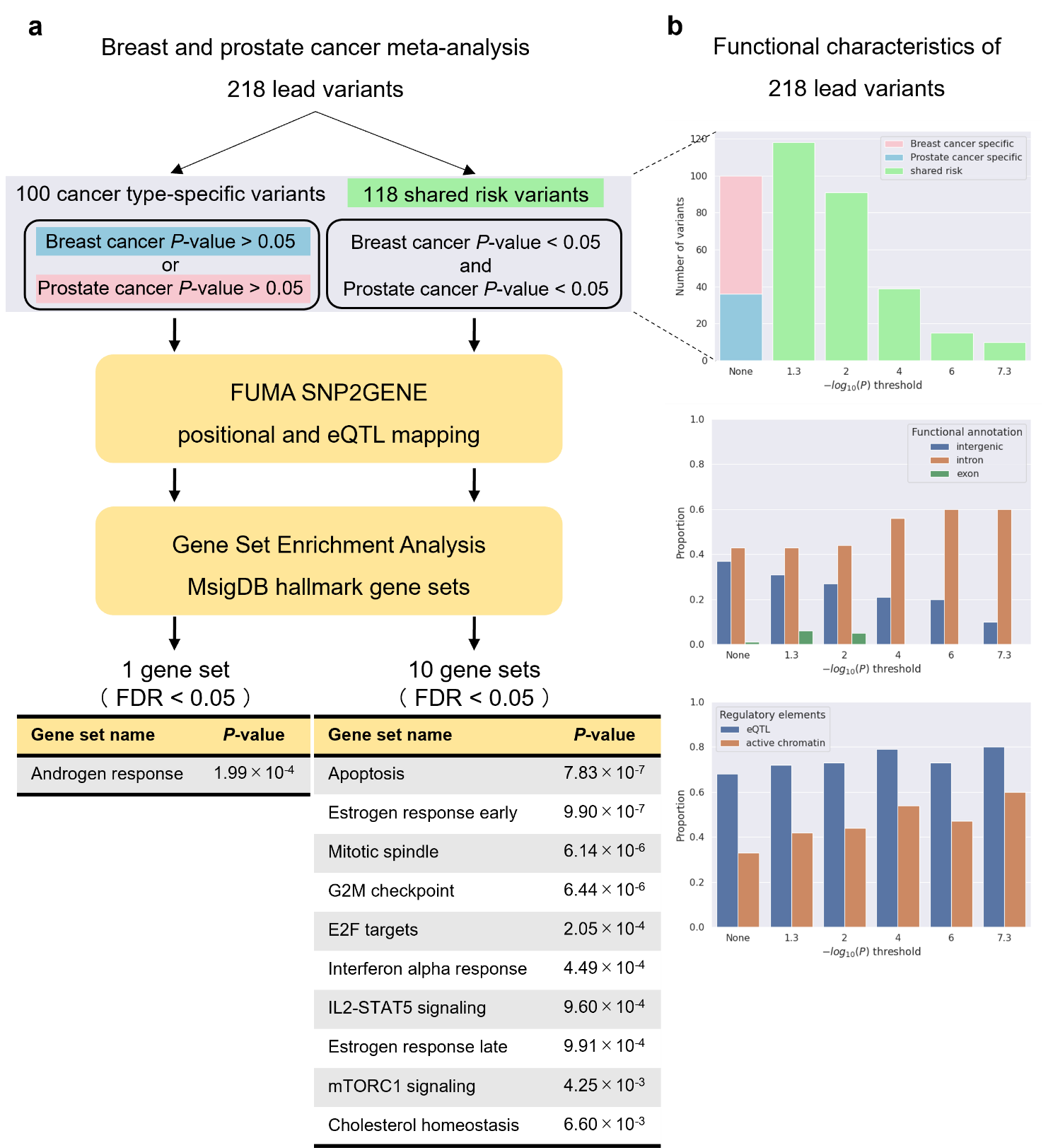
**Supplementary Fig. 11. Downstream analysis of the breast and prostate cancer large-scale meta-analysis.**

**a** Overview of the functional gene mapping and gene set enrichment analysis of the shared risk variants associated with both breast and prostate cancer and the cancer type-specific variants. *P*-values are uncorrected and reflect two-sided tests. FDR was calculated via the Benjamini-Hochberg method across all gene sets. **b** Functional characteristics of the cancer type-specific variants and the shared risk variants. The x-axis indicates the threshold of the associations between the lead variants and both breast and prostate cancer (1.3: the nominal threshold of *P* = 0.05; 7.3: the genome-wide threshold of *P* = 5.0 × 10^-8^).

**Supplementary Fig. 12. Breast cancer related gene expression levels in the breast cancer**
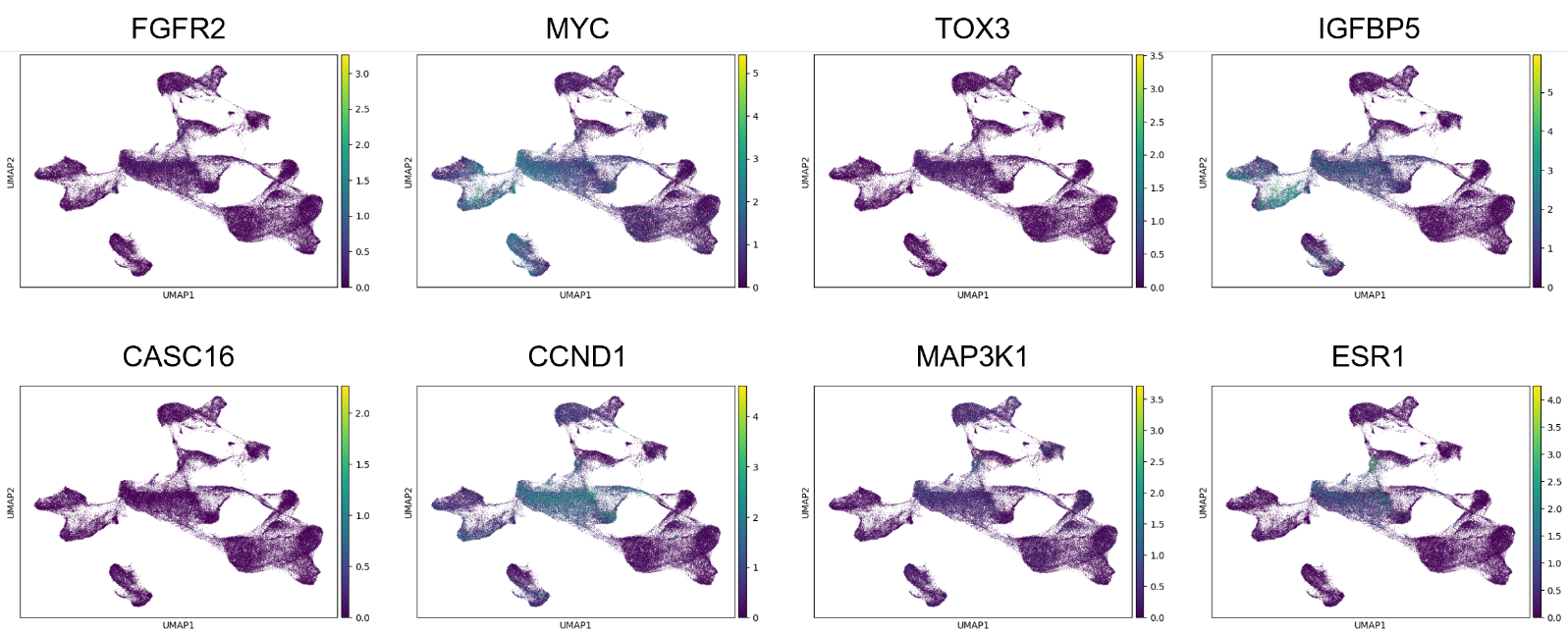
**single-cell RNA sequence dataset.**

UMAP visualizations of the breast cancer single-cell RNA sequence dataset colored according to expression levels of the breast cancer related genes.
