# Supplementary material for: Pan-cancer and cross-population genome-wide association studies dissect shared genetic backgrounds underlying carcinogenesis
Source: Nat Commun. 2023 Jun 20;14:3671. doi: 10.1038/s41467-023-39136-7 (PMC10282036; doi:10.1038/s41467-023-39136-7)
Supplement: Supplementary file 2 — Description of Additional Supplementary Files [file 41467_2023_39136_MOESM2_ESM.pdf]

Description of Additional Supplementary Files

File Name: Supplementary Data 1
Description: Genome-wide significant loci from the single cancer GWAS.

File Name: Supplementary Data 2
Description: Genome-wide significant loci from the single/all cancer meta-analysis.

File Name: Supplementary Data 3
Description: Replication analysis of the previous reported GWAS signals using the single cancer GWAS.

File Name: Supplementary Data 4
Description: Summary of the breast and prostate cancer large-scale meta-analysis.

File Name: Supplementary Data 5
Description: Genome-wide significant loci from the breast and prostate cancer large-scale meta-analysis.

File Name: Supplementary Data 6
Description: Summary of the variants newly satisfying the genome-wide significance threshold from the breast and prostate cancer large-scale meta-analysis.

File Name: Supplementary Data 7
Description: The associations between the summary statistics of the breast and prostate cancer large-scale meta-analyses and the MsigDB hallmark gene sets.

File Name: Supplementary Data 8
Description: The associations between the summary statistics of the breast and prostate cancer large-scale meta-analyses and the cell types in the single cell RNA-seq datasets.

File Name: Supplementary Data 9
Description: Summary of the time-to-event analysis investigating the variants detected from the single/all cancer GWAS/meta-analysis.
