# Supplementary material for: Pan-cancer and cross-population genome-wide association studies dissect shared genetic backgrounds underlying carcinogenesis
Source: Nat Commun. 2023 Jun 20;14:3671. doi: 10.1038/s41467-023-39136-7 (PMC10282036; doi:10.1038/s41467-023-39136-7)
Supplement: Supplementary file 4 — Reporting Summary [file 41467_2023_39136_MOESM4_ESM.pdf]

nature portfolio

Corresponding author(s): Yukinori Okada

Last updated by author(s): May 24, 2023

Reporting Summary

Nature Portfolio wishes to improve the reproducibility of the work that we publish. This form provides structure for consistency and transparency in reporting. For further information on Nature Portfolio policies, see our Editorial Policies and the Editorial Policy Checklist.

Statistics

For all statistical analyses, confirm that the following items are present in the figure legend, table legend, main text, or Methods section.

n/a	Confirmed	
☐	☒	The exact sample size (*n*) for each experimental group/condition, given as a discrete number and unit of measurement
☐	☒	A statement on whether measurements were taken from distinct samples or whether the same sample was measured repeatedly
☐	☒	The statistical test(s) used AND whether they are one- or two-sided<br>*Only common tests should be described solely by name; describe more complex techniques in the Methods section.*
☐	☒	A description of all covariates tested
☐	☒	A description of any assumptions or corrections, such as tests of normality and adjustment for multiple comparisons
☐	☒	A full description of the statistical parameters including central tendency (e.g. means) or other basic estimates (e.g. regression coefficient) AND variation (e.g. standard deviation) or associated estimates of uncertainty (e.g. confidence intervals)
☐	☒	For null hypothesis testing, the test statistic (e.g. *F*, *t*, *r*) with confidence intervals, effect sizes, degrees of freedom and *P* value noted<br>*Give P values as exact values whenever suitable.*
☒	☐	For Bayesian analysis, information on the choice of priors and Markov chain Monte Carlo settings
☒	☐	For hierarchical and complex designs, identification of the appropriate level for tests and full reporting of outcomes
☒	☐	Estimates of effect sizes (e.g. Cohen's *d*, Pearson's *r*), indicating how they were calculated

Our web collection on statistics for biologists contains articles on many of the points above.

Software and code

Policy information about availability of computer code

Data collection	No software was used in data collection.
Data analysis	We used the publicly available software for the data analysis (SAIGE0.43, RE2C, BOLT-LMM2.3.6 using a Monte Carlo algorithm, LDSC1.0.1, plink 1.9 and 2.0, Minimac3, Minimac4, IMPUTE4, LocusZoom (http://locuszoom.org/), ANNOVAR (https://annovar.openbioinformatics.org/en/latest/), LocusFocus1.4.9, PASCAL (https://www2.unil.ch/cbg/index.php?title=Pascal), FUMA1.3.8, GSEA4.2.3, scDRS1.0.0, MAGMAv1.10, and SPACox-0.1.2).

For manuscripts utilizing custom algorithms or software that are central to the research but not yet described in published literature, software must be made available to editors and reviewers. We strongly encourage code deposition in a community repository (e.g. GitHub). See the Nature Portfolio guidelines for submitting code & software for further information.

Data

Policy information about availability of data

All manuscripts must include a data availability statement. This statement should provide the following information, where applicable:

- Accession codes, unique identifiers, or web links for publicly available datasets
- A description of any restrictions on data availability
- For clinical datasets or third party data, please ensure that the statement adheres to our policy

GWAS genotype data of the BBJ are available at the NBDC Human Database (research ID: hum0014 and hum0311). All the GWAS summary statistics of our study are

publicly available at the NBDC Human Database (research ID: hum0197) and PheWeb.jp (https://pheweb.jp/) without restriction. The UKB analysis was conducted via application number 47821 (https://www.ukbiobank.ac.uk/). We used the FinnGen release 6 data. Summary results can be accessed through application at https://www.finngen.fi/en/access_results/. The summary statistics of BCAC and PRACTICAL are available at http://bcac.ccge.medschl.cam.ac.uk/ and http://practical.icr.ac.uk/blog/, respectively. The breast cancer scRNA-seq data are available for download through the Broad Institute Single Cell portal at https://singlecell.broadinstitute.org/single_cell/study/SCP1039. The prostate cancer scRNA-seq data have been deposited in the Gene Expression Omnibus (GEO) under accession no. GSE141445 and the Genome Sequence Archive for Human (GSA-Human) under accession HRA000312 and can be accessed at http://www.pradcellatlas.com/. The GTEx v8 and ImmuNexUT data were obtained from the GTEx portal (https://gtexportal.org/home/) and the ImmuNexUT website (https://www.immunexut.org/), respectively.

Human research participants

Policy information about studies involving human research participants and Sex and Gender in Research.

Reporting on sex and gender	We used the term sex as with other genomic studies. We collected clinical information including sex from medical records in BBJ. In UKB, we excluded subjects with mismatch between inferred sex from the genotype data and self-reported sex.
Population characteristics	All the Japanese subjects were included in BBJ, a prospective biobank that collaboratively collected DNA, serum samples, and clinical information in Japan. In BBJ, mean age of the participants at recruitment was 63.0 years old, and 46.3% were female. UKB is a population-based prospective cohort that recruited approximately 500,000 people across the United Kingdom. Mean age of the participants at recruitment was 56.8 years old, and 53.8% were female.
Recruitment	In the discovery GWAS/meta-analysis, all the Japanese subjects were included in BBJ1 (the first cohort of BBJ), that collaboratively recruited approximately 200,000 patients with ≥1 of 47 diseases in Japan between 2003 and 2007. The samples for the replication analysis were registered in BBJ2 (the second cohort of BBJ) between 2013 and 2018, which included approximately 80,000 new patients with 38 target diseases. For the European subjects, we extracted the subjects from UKB, a population-based cohort of approximately 500,000 people between 2006 and 2010 across the United Kingdom.
Ethics oversight	All the participants in BBJ provided written informed consent approved from ethics committees of the Institute of Medical Sciences, the University of Tokyo and RIKEN Center for Integrative Medical Sciences. This study was approved by the ethical committee of Osaka University Graduate School of Medicine.

Note that full information on the approval of the study protocol must also be provided in the manuscript.

Field-specific reporting

Please select the one below that is the best fit for your research. If you are not sure, read the appropriate sections before making your selection.

☒ Life sciences ☐ Behavioural & social sciences ☐ Ecological, evolutionary & environmental sciences

For a reference copy of the document with all sections, see nature.com/documents/nr-reporting-summary-flat.pdf

Life sciences study design

All studies must disclose on these points even when the disclosure is negative.

Sample size	All the sample sizes in the single cancer GWAS of the 13 cancers and the breast and prostate cancer large-scale GWAS meta-analysis are summarized in Table 1 and Supplementary Data 4, respectively. We did not perform sample size calculation but included the maximum number of individuals in each cohort who passed the QC threshold.
Data exclusions	All the samples in the single cancer GWAS of 13 cancers were selected based on the quality-control criteria in each cohort, which is summarized in the Method section.
Replication	We used the BBJ2 datasets including 31,712 cancer cases and 38,088 controls for replication. The sample sizes of each cancer are shown in Table 1. We evaluated the associations between the cancer risks and the variants identified from the discovery GWAS/meta-anslysis. We also conducted the combined meta-analysis across the discovery and replication datasets for all the variatns showing the suggestive associations in the discovery GWAS/meta-analysis including the BBJ1 datasets.
Randomization	We did not need to use randomization in this study because this is a genotype-phenotype association study and we used all of the recruited data.
Blinding	We did not apply blinding of the samples because this is a genotype-phenotype association study and no intervention was conducted in our study.

Reporting for specific materials, systems and methods

We require information from authors about some types of materials, experimental systems and methods used in many studies. Here, indicate whether each material, system or method listed is relevant to your study. If you are not sure if a list item applies to your research, read the appropriate section before selecting a response.

Materials & experimental systems

n/a	Involved in the study
☒	☐ Antibodies
☒	☐ Eukaryotic cell lines
☒	☐ Palaeontology and archaeology
☒	☐ Animals and other organisms
☒	☐ Clinical data
☒	☐ Dual use research of concern

Methods

n/a	Involved in the study
☒	☐ ChIP-seq
☒	☐ Flow cytometry
☒	☐ MRI-based neuroimaging
